# Automated Phenotyping Indicates Pupal Size in *Drosophila* Is a Highly Heritable Trait with an Apparent Polygenic Basis

**DOI:** 10.1534/g3.117.039883

**Published:** 2017-03-02

**Authors:** R. Guy Reeves, Diethard Tautz

**Affiliations:** Department of Evolutionary Genetics, Max Planck Institute for Evolutionary Biology, 24306 Plön, Germany

**Keywords:** automated phenotyping, *Drosophila melanogaster*, pupal length, human height, complex trait

## Abstract

The intense focus on studying human height has done more than any other genetic analysis to advance our understanding of the heritability of highly complex phenotypes. Here, we describe in detail the properties of a previously unexplored trait in *Drosophila melanogaster* that shares many salient properties with human height. The total length of the pupal case varies between 2.8 and 3.9 mm among natural variants, and we report that it is among the most heritable traits reported in this species. We have developed a simple semiautomatic phenotyping system with which a single operator can reliably score >5000 individuals in a day. The precision of the automated system is 0.042 mm (± 0.030 SD). All phenotyped individuals are available to be mated in subsequent generations or uniquely archived for future molecular work. We report both broad sense and narrow sense heritability estimates for two biologically distinct data sets. Narrow sense heritability (*h*^2^) ranged from 0.44 to 0.50, and broad sense heritability (*H*^2^) ranged from 0.58 to 0.61. We present results for mapping the trait in 195 recombinant inbred lines, which suggests that there are no loci with >10% effect size in this panel. We propose that pupal size genetics in *Drosophila* could represent a model complex trait amenable to deep genetic dissection using the automated system described.

Elucidating the genetic architecture of a phenotypic trait fundamentally requires that it is, to some degree, heritable. Phenotypic traits with low heritability do not generally require additional explanation as to why quantitative trait loci (QTL) cannot be robustly identified. However, while traits with high heritability increase the power to identify causative loci, the genetic architecture of traits remains a key factor in predicting success in identifying QTL. For example, a highly heritable trait that is dependent on a large number of interacting alleles or loci may still require substantial sample sizes and complex analyses to identify causative loci (Visscher *et al.* 2010; Barton *et al.* 2016). The archetypical example of this is human height, which, by at least two distinct measures of heritability, is among the most heritable of quantitative human traits, *h*^2^ ≈ 0.4–0.7 using regression of trios (Galton 1886; Hanley 2004; Visscher *et al.* 2010) or *h*^2^ ≈ 0.7 using twin studies (Polderman *et al.* 2015). However, the identification of the loci involved, and any interactions among them, has proven far from trivial, and successes to date have been dependent on utilizing very large data sets [up to 250,000 individuals (Wood *et al.* 2014)]. The current understanding being that of the 180–4000 loci implicated in impacting human height over its typical range all have additive effect sizes of <<1 mm (Weedon *et al.* 2008; Visscher *et al.* 2010; Wood *et al.* 2014). Although studies of human height represent a valuable model with which to test how complex heritable phenotypes can be dissected, the ability to experimentally control allele frequencies and environmental conditions would be a valuable capacity that is not possible in human studies. While this is a possibility in model organisms, there are relatively few morphological traits that have been robustly demonstrated to be both highly heritable and amenable to automated high throughput phenotyping (though see below). Herein, we describe in detail a new quantitative trait—length of the pupal case—in *Drosophila melanogaster* that places it in the 15–20% most heritable morphological traits described in this species, and is amenable to reliable and automated high-throughput phenotyping (Roff and Mousseau 1987).

The utility of automated phenotyping systems for a wide variety of organisms has increased greatly over the last 10 yr. While automated morphological phenotyping systems have been developed for *Drosophila*, as yet only automation analysis of images of wings has been widely employed (Houle *et al.* 2003). However, acquiring the wing images requires manual manipulation to position flies individually and so is difficult to scale up. Likewise, a system to measure heartbeat function requires that each fly be manipulated into position. An alternative approach to phenotyping in a selection experiment for gross body size was achieved using a series of graduated sieves (Turner *et al.* 2011). More recently, a sophisticated platform called the “fly cat walk” was described that has the capacity to reliably phenotype 700 flies a day for a wide range of morphological traits simultaneously (Medici *et al.* 2015). This requires no user manipulation of individual flies and is nondestructive. While the cost of constructing the equipment is not detailed (https://github.com/IMSB/FlyCatwalk/), it is likely that it would represent a significant investment of time, expertise, and resources for most laboratories.

Our setup uses an inexpensive camera in a light-proof box and the open source image analysis software Cellprofiler, with which a single user can phenotype 5000 pupae in a day. Pupae are photographed *in situ* on flattened squares of transparent film that lined the entire vertical surface of the vials. We demonstrate how the system can be used to (1) measure panels of recombinant inbred lines (RILs), and (2) generate large numbers of parent offspring trios that are particularly useful in exploring complex traits using artificial selection techniques. Furthermore, the increased throughput facilitates exploring the heritability of family means rather than single individual measurements, which are associated with increased measurement variance. Hence, pupal size could become a model phenotype that will allow deep dissection of its genetic architecture, since the availability of automated phenotyping will allow to screen very large mapping panels, or to design new complex mapping strategies.

## Materials and Methods

### Fly stocks

The automated phenotyping system was applied to two independent datasets.

The first dataset, referred to here as “eight-way” is a collection of 195 RILs that are part of the *Drosophila* Synthetic Population Resource (http://wfitch.bio.uci.edu/∼dspr/). These RILs are all originally derived from a cross between eight global stocks of *D. melanog*aster, their generation is described in detail in King *et al.* (2012a,b). Narrow sense heritability, *h*^2^, for the eight-way dataset was estimated by measuring the progeny of 67 single pair matings between individuals from 11 different RILs that spanned the full range of phenotype measurements (IDs of crossed stocks are given in Supplemental Material, File S5; six of the 67 crosses are duplicates also indicated in File S5). Broad sense heritability estimates *H*^2^ of the eight-way dataset were generated by repeated measurements of RILs (IDs given in File S5).

The second dataset, termed “four-way,” was initiated by a cross between two Japanese and two African stocks (see Table S1). Narrow sense heritability, *h*^2^, was estimated by measuring the progeny of single pair matings from the 2nd to 6th generation where phenotyped parents were selected randomly from different vials to form subsequent generations. The following number of single pairs were measured in each generation [G2, 15 pairs], [G3, 81 pairs], [G4, 78 pairs], [G5, 88 pairs], and [G6, 154 pairs]. No duplicate vials were generated from the same mating pair. Note that, unlike the eight-way dataset, levels of heterozygosity in the parents and their offspring are likely to be similar. *H*^2^ estimates of the four-way dataset were generated by measuring 83 RILs established from the four-way cross by generation 37 (see File S5 for stock IDs). Details of all progenitor stocks of the four-way, and four-way datasets with estimates of their pupal length, are given in Table S1.

### Image acquisition

Flies were maintained on standard food dispensed into 28.5 mm diameter, 95 mm height vials (Genesee Scientific). Once the food vials had fully cooled, 10 cm × 10.5 cm squares of overhead projector film were slid into each vial lining their entire vertical wall (nobo, plain paper copier film, 33638237). A more detailed description of the entire procedure and equipment set up is provided as File S1. A custom printed semitransparent label, including a unique barcode, was affixed to the outside of each vial. Vials were incubated at 24° in a 12 hr light/12 hr dark incubator. Adults were removed after one to two nights in vials (sometimes three to four nights, if fertility appeared to be low, or due to holidays). Generally, by the 10th day after the parents were initially introduced, the majority of offspring in the vials were present as pupae attached to the transparent film; few if any larvae remained in the food. The film from each vial was removed, and placed into a purpose-made plastic frame (this frame can be 3D printed using a file provided as File S2) that holds the film flat for photographing. Food from the lower part of the film was scraped away and any larvae, or any white puparium stage (P1), removed. The frame was then photographed using bottom illumination in a light tight box. Batches of the resulting images were then analyzed using the procedure below.

### Automated image analysis

A Cellprofiler (v2.1.0) pipeline was developed to simultaneously recognize pupae and to measure a variety of attributes, including length. The outputs of all measurements are written to .xls files that can then be viewed or imported into any database program. Cellprofiler is free, open access, software providing a suite of flexible image analysis tools (Lamprecht *et al.* 2007). In brief, the Cellprofiler pipeline first identifies “primary objects” distinct from the background without restriction on their size (module: identify primary objects). Then, applying a scalable model of pupal shape to all objects, those that are composed of multiple touching pupae are separated into distinct pupae [module: Untangle Worms, (Wählby *et al.* 2012)]. The resulting putative pupae are then each shrunk, and then repropagated outwards to more precisely identify the edges of each pupa based on boundary changes in pixel intensity (module: Identify Secondary objects). Finally, pupae are crudely filtered on size attributes and the proximity of neighboring objects to place them in one of the three confidence classes described in the *Results*. The digital outlines of pupae are overlaid onto a cropped version of the original image to allow users to easily visually assess quality.

Overlaid images and files of measurements were imported in batches into the database program FileMaker (v14, FileMaker Inc.). A unique barcode sticker identifying the film in the image was automatically read by the database. This enabled the image files to be automatically renamed with the barcode as their name for archiving. In addition to pupal measurements, a 1 €cent coin (16.25 mm diameter) present in all images was measured to control for camera changes, and to allow conversion of measurement in pixels to millimeter. Quality filtering of pupae and basic analyses were performed using the database. An annotated copy of the Cellprofiler pipeline is provided as File S3.

### Estimates of heritability

*H*^2^ was estimated using SPSS version 22 with the “variance components” function, with pupal length as the dependent variable, and RIL name as a random factor. This was done using the Minimum Norm Quadratic Unbiased method (though ANOVA produced identical results to two decimal places). The model for the single factor was *y_ij_* *=* *μ* + *α_j_* *+e_ij_*, where *y_ij_* is the *j*th observation of the *i*th RIL, *μ* is the overall mean, *α_j_* is the random factor, and *e* is the associated error.

Estimates of *h*^2^ were made using the “linear regression” function, with pupal length as the dependent variable, and parent-midpoints as an independent variable. All other statistical analyses and graphs were also performed using SPSS or Filemaker. All data for individual pupa are available as File S4, and for vial means as File S5 (fields used in each graph are indicated in the associated “readme” files).

### Human height data

Estimates of *h*^2^ for human height data were made using the same methods as for pupae, using the original data of Francis Galton for 898 individuals, transcribed from his 1880s laboratory notebooks (Hanley 2004) (available http://www.math.uah.edu/stat/data/Galton.html). The 760 individuals in 123 families where four or more individuals were measured were used for estimates of the *h*^2^ heritability of mean family height (females were not transmuted into males).

### Genome scan for loci of large effect impacting pupal length using eight-way RILs

Scans were performed in R (version 3.2.2) for the 195 DSPR RILs mentioned above (King *et al.* 2012a,b) using the “DSPRscan” command within the DSPRqtl-tools procedures described in King *et al.* (2012a,b). These utilize one SNP per 10 kb throughout the genome reference (with the exception of the Y chromosome and the mitochondrial genome). Significance LOD score thresholds were estimated using the “DSPRperm” command with 1000 replicates. The data input file is available as File S6. This scan was undertaken to asses whether one or few loci of large effect control pupal length, *i.e.*, the trait is not complex. Note that, if the trait is controlled by many loci of small or moderate effect size, the scan is too underpowered to realistically identify individual loci. However, the goal of the scan is to show that pupal size is not a relatively simple trait controlled by only a few major effect loci.

### Data availability

All starting fly stocks are available from the sources detailed in Table S1. RILs are available from the web address given above. Files of raw data are available as File S4, File S5, and File S6. 

## Results

**Figure 1:**
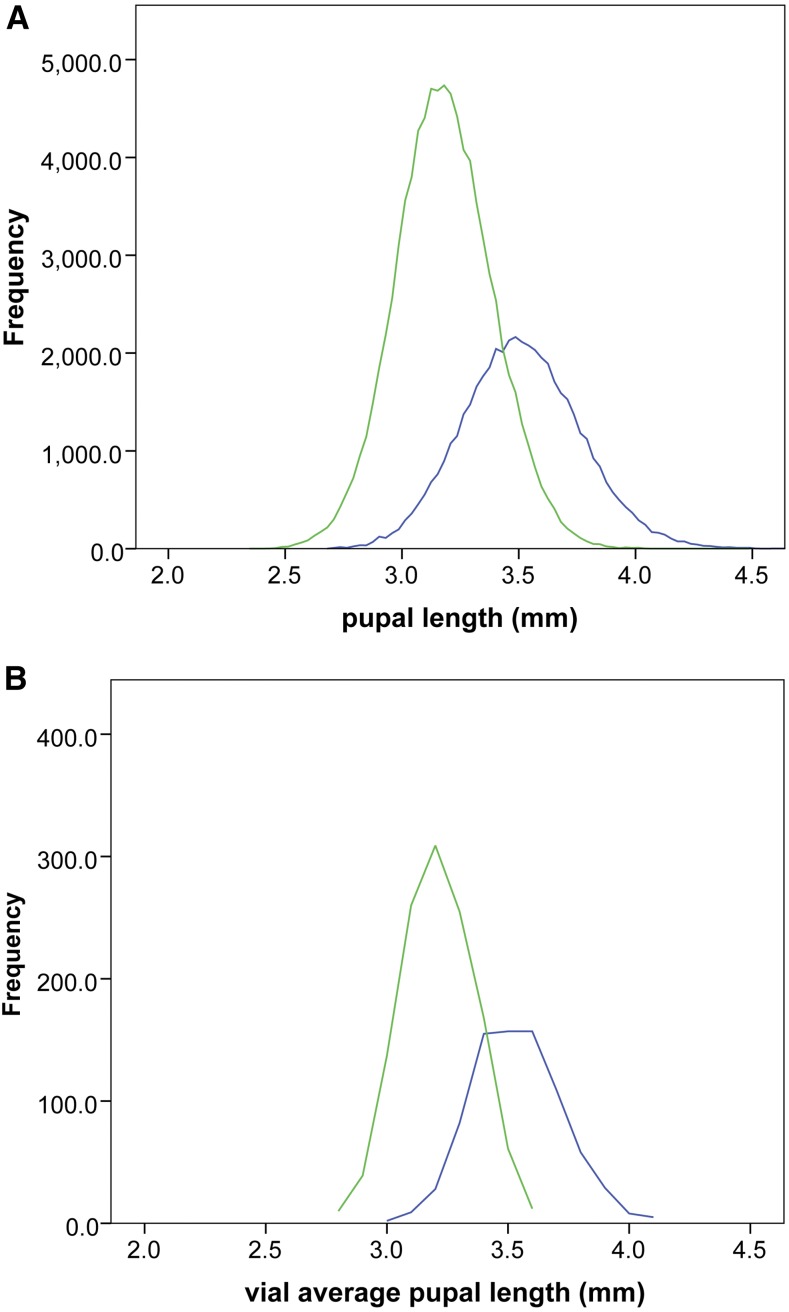
Sampling distributions of pupal length for individuals and vials. (A) Distributions for individual pupal lengths: four-way dataset (blue) *n* = 47,843 and eight-way dataset (green) *n* = 86,515. (B) Corresponding distributions for vial mean pupal lengths: four-way dataset (blue) *n* = 799 vials and eight-way dataset (green) *n* = 1251 vials. Only vials where ≥15 pupae were measured by the automated system are considered. A stock possessing the Tb^1^ mutation, resulting in the well-known tubby pupal phenotype (Bloomington stock 3644), was measured and found to have a mean pupal length of 2.7 mm, which is at the lower bounds of the smallest wildtype individuals or vials shown here.

The large numbers of individuals and vials measured, made possible through the use of the automated measuring system, provides robust insights into how phenotypic variation is partitioned for pupal length. While the method automatically identifies and measures pupae for multiple different parameters (see File S1 for a full list), the length of the pupae was the principle focus of this study. Two distinct sets of biological material were used to define the biological properties of pupal length. The first dataset was all derived from an eight-way cross (King *et al.* 2012a,b); the second was from a four-way cross. None of the founding stocks are shared between the eight-way and the four-way datasets. *H*^2^ (reflecting all potentially genetic contributions to trait variance *e.g.*, additive, epistatic, dominance, maternal, and paternal effects) was estimated through the analysis of repeated measurements of RILs, whereas *h*^2^ heritability (reflecting only the impact of additive genetic effects) was measured by regression of midparent against their offspring measurements. Sampling properties of the eight-way and four-way datasets are detailed in Table 1 and Figure 1 (see also Figure S1 for summary of repeat measurements of RILs).

**Table 1:** Summary of sampling of eight-way and four-way datasets

Dataset	Heritability	Vial	Individuals
Eight-way	*H*^2^	Vials measured = 1184, RILs = 195	*n* = 83,402
		Mean replicates per RIL= 6.7 (± 2.4 SD, min = 4, max = 15)	Mean pupal length = 3.2 (± 0.21 SD)
		Mean vial pupal length = 3.2 (± 0.15 SD)	
		Range of 95% of pupal lengths = 2.8–3.5 mm	
		Mean number of measured pupae per vial = 70 (± 34 SD)	
	*h*^2^	Crosses measured = 67	*n* = 3113
		Mean vial pupal length = 3.5 (± 0.11 SD)	Mean pupal length = 3.4 (± 018 SD)
		Range of 95% of pupal lengths = 3.1–3.5 mm	
		Mean number of measured pupae per vial = 47 (± 15 SD)	
Four-way	*H*^2^	Vials measured = 436, RILs = 81	*n* = 25,356
		Mean replicates per RIL = 5.54 (± 0.78 SD, min = 3, max = 6)	Mean pupal length = 3.6 (± 0.24 SD)
		Mean vial pupal length = 3.6 (± 0.15 SD)	
		Range of 95% of pupal lengths = 3.4–4.0 mm	
		Mean number of measured pupae per vial = 59 (± 24 SD)	
	*h*^2^	Crosses measured = 363	*n* = 22,487
		Mean vial pupal length = 3.4 (± 0.14 SD)	Mean pupal length = 3.4 (± 0.23 SD)
		Range of 95% of pupal lengths = 3.1–3.7 mm	
		Mean number of measured pupae per vial = 62 (± 17 SD)	

Only vials where ≥15 pupae were measured by the automated system are considered. For *H*^2^ estimates, only RILs where ≥3 replicate measurements were available are considered.

### Performance of automated pupal phenotyping

The automated pupal recognition pipeline attempts to identify the external outlines of three classes of objects:

*High confidence pupae*: conform to a set of expected properties and that have no neighboring pupae within 20 pixels (≈0.7 mm, Cellprofiler module: filter objects)
*Medium confidence pupae*: conform to the same set of properties but with neighboring objects closer than 20 pixels. This often occurs as larvae select pupation sites touching each other, resulting in a more challenging target for image recognition
*Low quality objects*: unlikely to be pupae.

**Figure 2:**
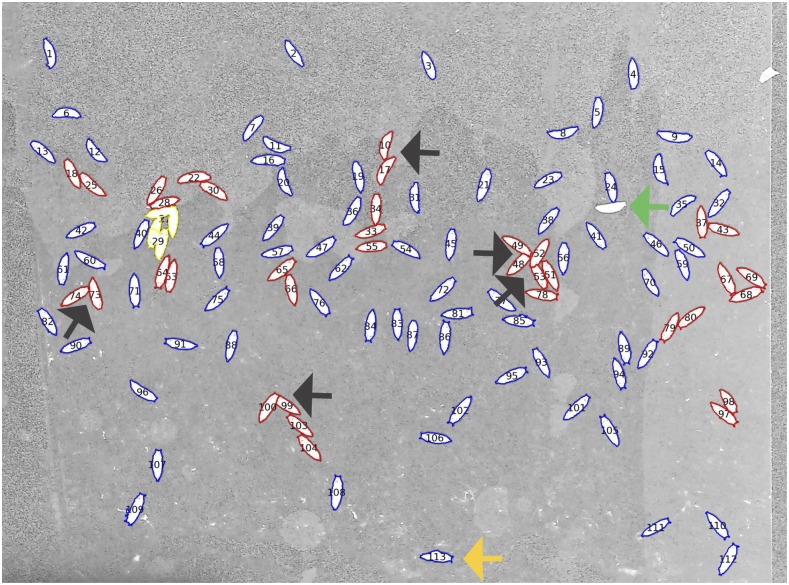
Visual output of automated phenotyping system. Blue outline = high confidence pupae, red outline = medium confidence pupae, and yellow outline = low quality objects. A small proportion of pupae are entirely missed by the automated system (green arrow). Others are aberrantly measured due to close proximity to other pupae resulting in truncation or extension of their outline. Even those with a high confidence assignment can also appear malformed (orange arrow). They should be manually excluded from analysis to reduce noise (see File S1 for more examples of aberrant and canonical pupae). All objects are numbered in Cellprofiler output files to permit numerical measurements to be easily related to images.

Examples of the three classes are shown in Figure 2 (see File S1 for further details).

Of the pupae retained for analysis, 47 and 53% were high and medium confidence, respectively. The probability of pupae being manually excluded as aberrantly measured was 0.02 for high quality pupae and 0.10 for medium confidence pupae (Figure 2). Note that not all images were examined manually, as, due to the robustness of the automated estimates, generally only vials with atypically high variance of the mean were examined.

**Figure 3:**
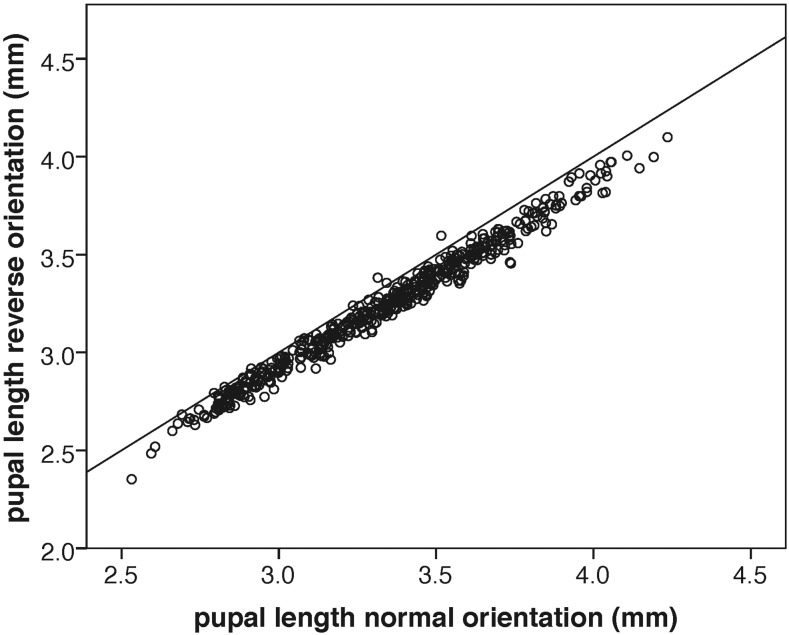
Precision of automated pupal length estimates. Comparison of pupal length measurements of films in the normal orientation compared to where the same film was rotated by 180° All pupae isolated from others by up to 15 pixels in any direction were used (*n* = 516 pupae, across 10 vials). Slope = 0.94 and *R*^2^ = 0.98. An *x* = *y* line is shown for reference.

The precision of the automated system was assessed by remeasuring a subset of films after rotating them by 180°, and comparing 516 duplicated measurements of the same pupae (Figure 3). The difference between the two measurements was an average of 0.043 mm (±0.030 SD). Remeasuring pupae after delays of 15 or 30 hr also generated similar precision estimates, indicating that, once pupae become brown puparium (P2), there is no detectable change in pupal length. Furthermore, the exuvia of eclosed individuals can also be reliably measured (data not shown).

### The count of automatically measured pupae is a reasonable proxy for density within vials

**Figure 4:**
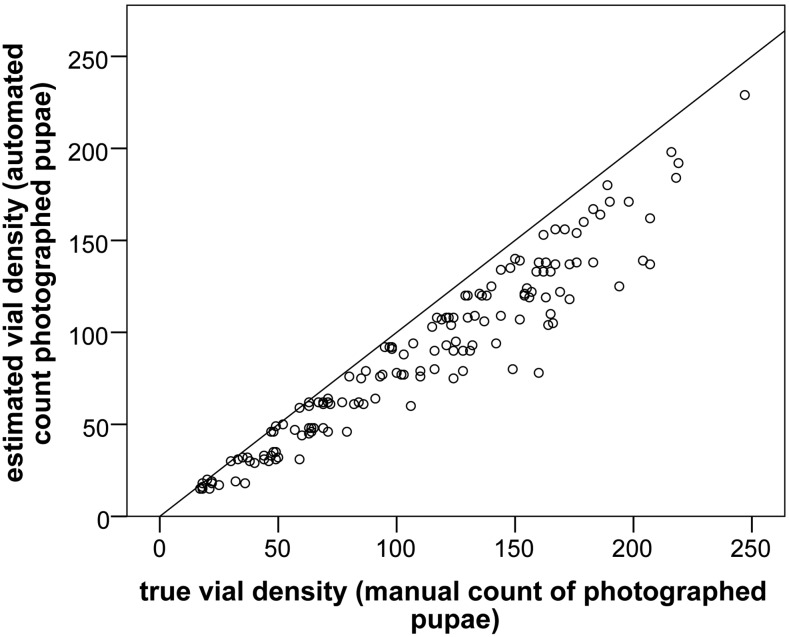
Comparison of automated count of pupae per vial *vs.* manual counting; 148 vials from across the entire range of the automated densities observed were manually recounted. While at higher densities the accuracy of the automated count decreases, it remains a reasonable proxy for vial density across the entire observed range. Slope = 0.81 and *R*^2^ = 0.92. An *x* = *y* line is shown for reference.

Unsurprisingly, not all pupae were correctly captured by the automated system, and, based on a sample of 148 vials, an average 20% ± 11.7 SD pupae per vial were missed, called as low confidence objects, or manually excluded (Figure 4 and Figure 2). While there is an increased variance in the proportion of unmeasured pupae in higher density vials (*e.g.*, where the automated count is >80), even here the automated count provides a reasonable proxy for density of individuals in each vial. This assumes that few, if any, larvae remained on the food surface after film transfer, as was generally the case (due in part to the short period parents remained in the vials). In addition, the small number of pupae removed from films that were obscured by larval food represents a constant proportion across all vials (see File S1).

### Impact of vial density on pupal length

One common major environmental covariate of many *Drosophila* traits is density of individuals within the vial in which they develop. Consequently, many *Drosophila* researchers control for this in experiments by collecting large numbers of zygotes and placing a controlled number in each vial. This is a fairly laborious process to perform routinely, and is complicated considerably where single pair crosses are required.

**Figure 5:**
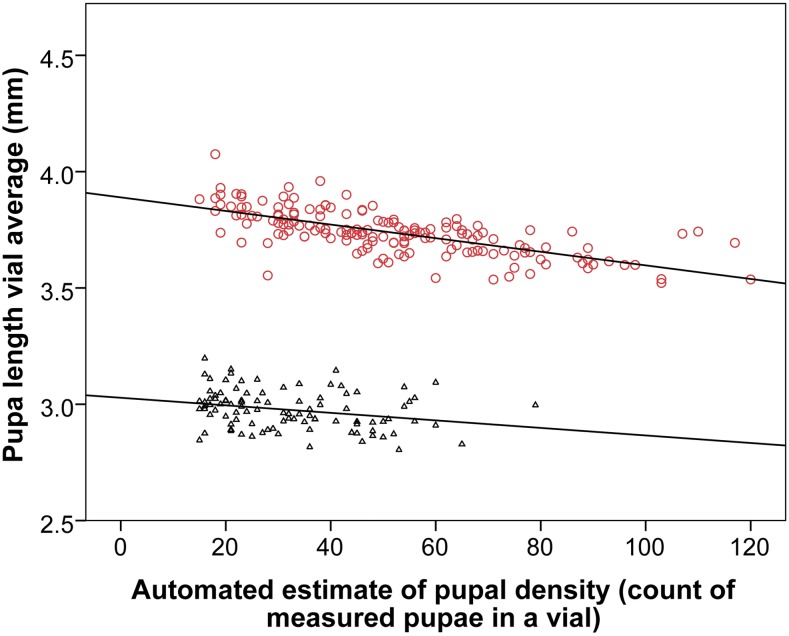
Relationship between vial density and pupal length for the two control stocks. Repeated measurements of the same stocks (one short and one long) at different densities permits the slope and correlation of the relationship to be estimated. While there is a high degree of correlation for the longer stock-329 (red circles) (*R*^2^ = 0.48 and *P* = >0.001), the correlation for the shorter stock-335 (black triangles) is minimal (*R*^2^ = 0.07 and *P* = 0.07).

Here, density was controlled only indirectly through limiting the number of parents used per vial, and restricting the number of nights they remained before being cleared [generally two nights for single pair crosses, and one night for small groups (*n* = 10–20) of RIL individuals, see File S1]. Throughout all experiments, two stocks were continually remeasured to act as controls (stock 335 and 329, see Table S1). The large number of repeat measurements of these two stocks across a range of densities permits the examination of any relationship between density and pupal length (Figure 5). Furthermore, in the same way, it is also possible to explore the relationship between density and pupal length using the repeated eight-way and four-way RIL measurements (see Figure S2). All RILs and control stocks exhibit a uniformly negative relationship between density and pupal length; the slope varies from −0.0006 to −0.0041. This observed variability may reflect either variance in estimating slopes, or also that RILs exhibit different reaction norms. If the mode slope of −0.002 (Figure S2) is used to correct the mean length of vials (or individuals) to that observed at the mean observed vial density, this can be achieved with the following equation:

$$\begin{array}{l} M=\text{mean}\;\text{vial}\;\text{density}\;\text{across}\;\text{whole}\;\text{experiment}\\ S=\text{slope}\;\text{of}\;\text{regression}\;\text{of}\;\text{density}\;\text{against}\;\text{mean}\;\text{vial}\;\text{length}\left(-0.002\right)\\ D=\text{automated}\;\text{estimate}\;\text{of}\;\text{density}\;\text{in}\;\text{vial}\;\text{to}\;\text{be}\;\text{corrected}\\ Q=\text{individual}\;\text{length}\;\text{measurement}\;\text{or}\;\text{vial}\;\text{mean}\;\text{to}\;\text{be}\;\text{corrected}\\ \left[\left(D-M\right)S\right]+Q=\text{Pupal}\;\text{length}\;\text{corrected}\;\text{for}\;\text{vial}\;\text{density} \end{array}$$ (1)

**Figure 6:**
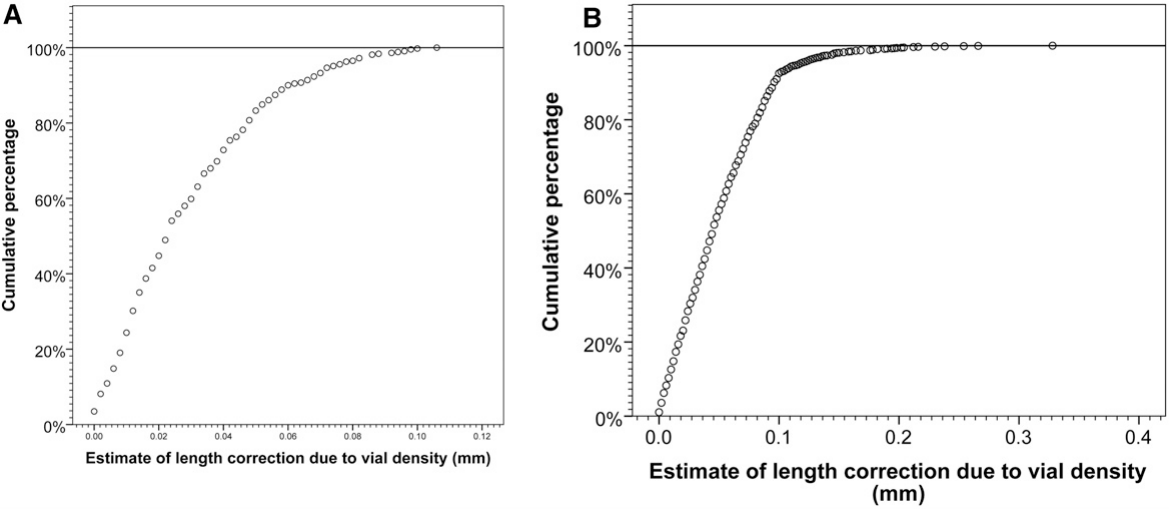
Magnitude of potential correction of pupal length for vial density, using Equation 1. The more uniform vial densities from single pair matings (A) resulted in all but one vial being within 50 pupae of the mean observed density of 65, corresponding to a maximum correction for density of 0.1 mm (*n* = 431 vials). The increased variance of the vial densities resulting from establishing vials from small groups of RIL individuals (B) led to ∼5% of vials being corrected by >0.1 mm (*n* = 1620 vials). A calculated mean density (*M*) of 65 was used for all vials. A slope value of *S* = −0.002 was used, if a steeper slope is used then there would be a corresponding increase in the magnitude of the corrections. All vial with a density of <15 were excluded.

Applying this formula to the 431 vials established as single pair crosses, >99% of them would require a correction of <±0.1 mm, and, of the 1620 vials established from small groups of RIL individuals, 95% would require a correction of <±0.118 mm (Figure 6). Given the modest number of vials subjected to a large correction, and uncertainly about whether there is a truly universal linear relationship between density and pupal length, none of the measurements presented in the manuscript have been corrected analytically for density. Furthermore, with respect to repeated RIL measurements, reducing any confounding impact of density can be achieved by either experimentally increasing the number of replicate measurements closer to the mean density, or, analytically, by excluding or weighting down vials with extreme density values.

### Variance in the estimates of vial means

**Figure 7:**
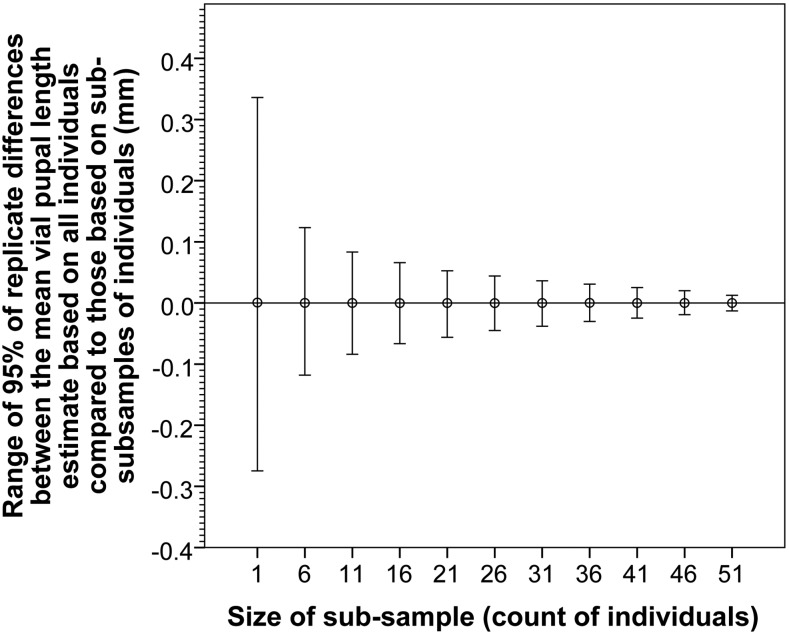
Deviation of mean vial pupal length of random subsamples compared to that based on all individuals within a vial. Each vial was resampled selecting only a subsample of the pupal length measurements within a vial to calculate a subsample mean length that was then subtracted from the mean length based on all sampled individuals. Each vial was resampled 100 times per subsample size. Whiskers represent range of 95% of subsample deviations, and circles the mean deviation of 100 replicates. Based on 729 vials, with densities ranging from 51 to 60 measured pupae.

A script was written in Filemaker to explore the extent to which estimates of mean vial pupal length based on subsamples of individuals within a vial deviate from the vial means based on all individuals. This provides insight into at what point vials with low densities generate mean estimates with unacceptably high variance. Figure 7 indicates that selecting single individuals to estimate vial means unsurprisingly results in a high degree of variance of up to 0.4 mm. While these estimates are unbiased, this is likely to prove unacceptably high given that the total observed range of pupal lengths is 1.1 and 0.8–0.9 mm within datasets. However, for random subsample sizes equal to a density of ≥15, the variance is greatly reduced to <0.07 mm for 95% of vials (Figure 7). On this basis, only vials with >15 measured pupae were included for analysis or presentation throughout this study.

### The impact of using smaller numbers of vials to estimate RIL mean length

**Figure 8:**
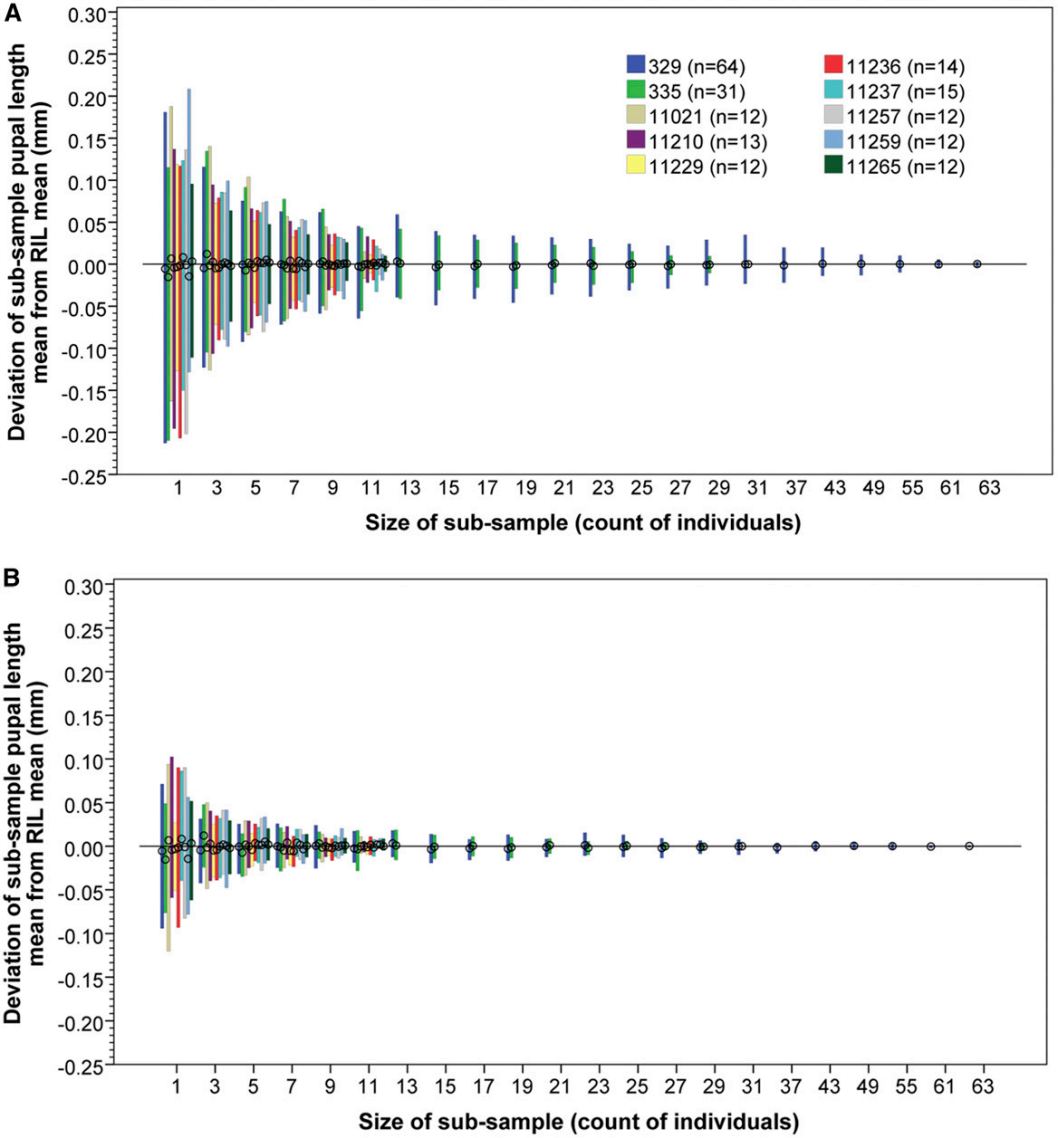
Deviation of mean RIL pupal length utilizing random subsamples of vials compared to that based on all measured vials. For all eight RIL, and two control stocks with 12 or more vial replicate measurements, the RIL mean pupal length was recalculated using a random subsample of vials. Each RIL was resampled 100 times per number of vial subsample size used. Whiskers represent range of subsample means, circles represent the overall means. (A) Range of 95% of subsamples; (B) 50% of subsamples. Total number of replicate vial measurements per RIL were as follows: RILs 11,229, 11,257, 11,259, 11,265, and 11,021 = 12 replicates; 11,210 = 13 replicates; 11,236 = 14 replicates; 11,237 = 15 replicates; 335 = 31 replicates; and 329 = 64 replicates. Note all pupae in vials were used to calculate each vial length mean, only the number of vial means in each subsample was varied in estimating length RIL means.

In measuring panels of RILs, it is useful, for practical reasons, to minimize the number of replicate vials measured for each RIL to estimate an RIL mean with an acceptable degree of variance. Using eight RILs and the two control lines, all of which were measured 12 times or more, it is possible to explore the relationship between RIL means and the number of replicate vial measurements used. Figure 8 indicates that six replicated measurements generally result in 95% of RIL estimates being <0.1 mm different from that based on larger numbers of replicate vial measurements. The mean number of replicate vial measurements per RIL in this study was 6.4 ± 2.1 SD (5.8% = the minimum four replicates, 18.0% = five replicates, 61.2% = six replicates, and 15% more than six replicates).

### Estimates of h^2^

**Figure 9:**
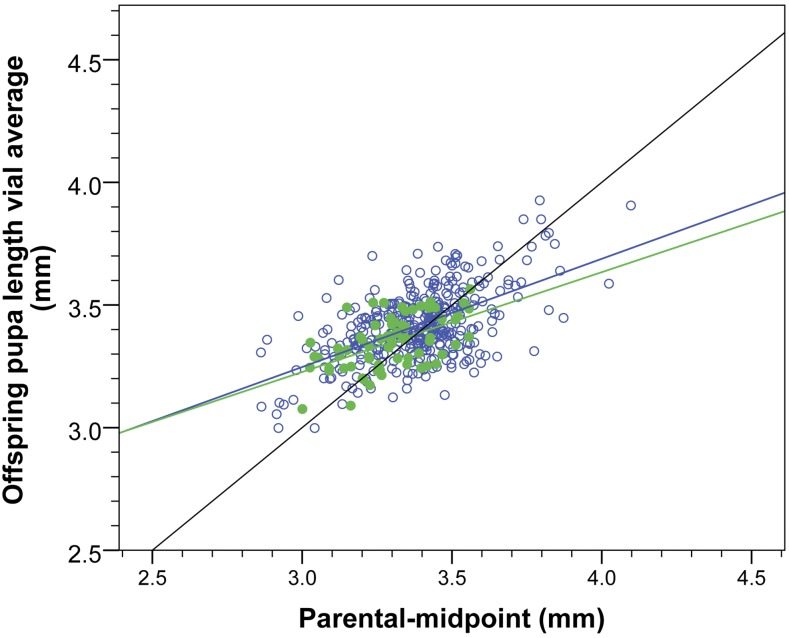
Estimates of mean vial length narrow sense heritability *h*^2^ for eight-way and four-way data sets. Despite no overlap in the stocks used to initiate both datasets, there is a remarkable similarity in the slope (*h*^2^) and the degree of correlation (*R*^2^) among them (see Table 2 for all parameter estimates). Green closed circles, eight-way crosses; blue open circles, four-way crosses.

Estimates of the additive genetic impact on the variance of the trait can be gained from the slope of regressing the parental midpoint [(length of father + length of mother)/2] against the length of the progeny (Galton 1886). Figure 9 indicates that, despite the eight-way (67 crosses) and four-way datasets (363 crosses) having no overlap in biological material, and having quite distinct sampling properties, they both result in remarkably similar estimates of *h*^2^, when representing all offspring of a cross as a mean. If all offspring are represented individually, then the estimate of heritability is largely unchanged for both datasets (Table 2). This robustness in the estimates is shared with human height (Table 2 and Hanley 2004). Likewise, regressions that use only the father or mother measurements, rather than their midpoint, confirm that paternal and maternal effects are of equal magnitude for both pupal length and human height (see Figure S3).

**Table 2:** Summary of heritability estimates for pupal length and human height

	Four-Way	Eight-Way	Human Height
*h*^2^ (mid-parent regression)	Offspring vial means	Offspring vial means	Offspring family (≥4) means
	0.44 ± 0.04 SE.	0.50 ± 0.09 SE.	0.68 ± 0.09 SE.
	*R*^2^ = 0.31	*R*^2^ = 0.33	*R*^2^ = 0.31
	Offspring individual	Offspring individual	Offspring individual
	0.42 ± 0.08 SE.	0.54 ± 0.02 SE.	0.68 ± 0.06 SE.
	*R*^2^ = 0.10	*R*^2^ = 0.14	*R*^2^ = 0.11
*H*^2^	Vial means 0. 58	Vial means 0. 61	Not possible
Expected *H*^2^ assuming additive only model (2*h*^2^)/(1 + *h*^2^)*^a^*	Vial means 0.61	Vial means 0.71	—

Data for both human and *Drosophila* is uncorrected for any sexual dimorphism.

a Mackay (2013).

### Estimates of H^2^

Estimates of all potentially genetic (*H*^2^) impacts on the variance of the trait can be obtained by estimating the proportion of the total variance in mean vial length measurements related to RIL stock. Table 2 summarizes the results with both the eight-way (195 RILs) and four-way (81 RILs) datasets generating very similar estimates of *H*^2^ of 0.58 and 0.61, respectively. The significance of ANOVA tests reflects the strong genetic signal eight-way *P* = 2.7 × 10^−140^ (*F* = 10.86, Mean Square = 0.093, d.f. = 194, sum of squares = 18.3) four-way *P* = 8.8 × 10^−50^ (*F* = 9.026, Mean Square = 0.087, d.f. = 80, sum of squares = 6.9).

### Whole genome scan for loci impacting pupal length

**Figure 10:**
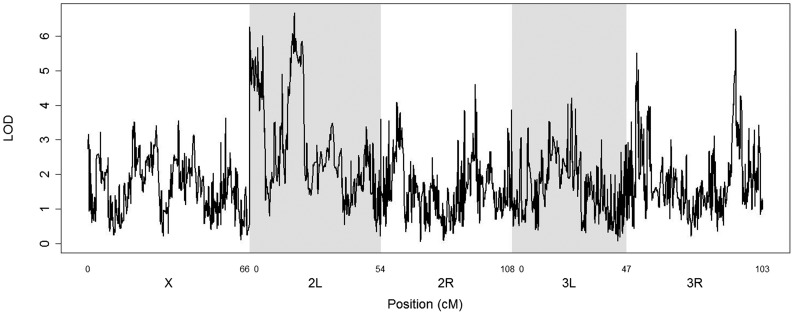
Genome scan of RIL pupal length in eight-way dataset (195 RILs means uncorrected for vial density). No peaks are significant at a *P* = 0.05 threshold of 7.02 LOD (estimated by permutation).

Using 195 RILs from the eight-way dataset, and the DSPR tools, a genome scan for genomic regions associated with mean RIL pupal length was conducted (unweighted mean of replicate vial means). The density of markers used in the analysis is one SNP per 10 kb across almost the entire reference genome (excluding the mtDNA and Y chromosome). No SNPs of significance were identified at *α* = 0.05; this was also the case if density corrected RIL means were used (Figure 10).

## Discussion

While (Visscher *et al.* 2010) stated that “One could argue that height in humans is the equivalent of bristle number in *Drosophila*, in terms of its role as a model phenotype.” we propose that pupal length is as good, if not a better, analog, based on its biological properties described in detail for the first time here. This is in addition to the capacity to automate phenotyping using a reliable low cost system. Heritability is estimated using two distinct measures, broad sense (*H*^2^) and narrow sense (*h*^2^), for both of two biologically independent datasets. This allows the consistency of heritability estimates to be assessed between two datasets, which likely span most of the range of the trait within *D. melanogaster*. Furthermore, the contrast between *h*^2^ (additive genetic effects only) and *H*^2^ (additive + nonadditive effects) permits the magnitude of nonadditive effects to be estimated. The properties of pupal length established in this manner are potentially salient to efforts to develop resource efficient means to dissect genetically complex traits, and similar to those reported for human height (Table 3). The key parameter being the high and consistent heritability estimates, which, in part, reflect the reliability of automated measurements where the estimated precision of measurements of 0.04 mm is small relative to the range of the trait as a species (1.1 mm), or within datasets (0.8–0.9 mm). The reliability of pupal length measurements also likely contributes to the normality of the distributions observed at every hierarchical level in the datasets (*e.g.*, Figure 1).

**Table 3:** Salient properties of pupal length relative to human height

	Adult Human Height	*D. melanogaster* Pupal Length (Based on this Study)
Relative heritability of trait	Among the most heritable reported in humans *h*^2^ = 0.4–0.7*^a^*	In top 80% of most heritable traits in *D. melanogaster h*^2^ = 0.4–0.5*^b^*
Measurement method	Manual	Automated
Typical range within species (ignoring sexual dimorphism)	≈70 cm	≈1.1 mm
Measurement error	≈1.5 cm*^c^*	0.042 mm (± 0.030 SD)
Ratio of measurement error: typical range of trait in species	≈0.02	≈0.04
Paternal and maternal impact on offspring trait variance.	Equal	Equal
Number of loci estimated to impact variance of trait	≈180–4000*^d^*	Unknown
Experimental manipulation of allele frequencies	Not possible	Easy

a Hanley (2004) and Visscher *et al.* (2010); corrected for sexual dimorphism.

b Not adjusted for sexual dimorphism.

c Voss *et al.* (1990).

d Aulchenko *et al.* (2009), Vinkhuyzen *et al.* (2013), and Wood *et al.* (2014).

The ease of phenotyping large numbers of individuals at a developmental stage where they are obligatorily stationary for 2–3 d has enabled robust insight into the heritability of pupal length at 24°. The very similar estimates (Table 2) of heritability for two independent datasets provides increased confidence that this trait represents an excellent prospect with which to attempt to identify the genes underlying this complex trait. Furthermore, the similarity between the observed *H*^2^ estimates and those expected based on a purely additive model of inheritance (see last row in Table 2) indicates that epistatic, dominance, and paternal or maternal effects are relatively modest (Mackay 2013). The observed similarity in the two *h*^2^ estimates (and the associated correlation *R*^2^) between the four-way and eight-way datasets (Figure 9) could also be argued to reflect a modest role for dominance. This is because, while the parents and offspring of the four-way crosses have similar levels of heterozygosity, the eight-way parents are inbred RILs while their offspring are heterozygous. Under some forms of dominance effects, both the slope and the correlation of the regression could be reduced in the latter case, which is not the case for pupal length. With respects to maternal and paternal effects, the data presented here (Figure S3) provide evidence that any nonadditive paternal or maternal effects are small, as the size of the effect mothers and fathers exert on their offspring is symmetrical [which is also a property of human height (Hanley 2004)].

It has been detailed above that the impact of density within vials on pupal length is relatively modest (mostly <0.1 mm, Figure 5 and Figure 6), and can be conveniently controlled for. While other environmental variables were not systematically explored, limited data collected for the two control lines indicates that between 18° and 24° pupal length changes by 0.2–0.4 mm (Figure S4), with pupal length being greater at cooler temperatures. The correlation between adult body weight and pupal length was also briefly examined and found to be *R*^2^ = 0.6 for both males and females (see Figure S5). Any correlation between adult body length and pupal length was not examined, though it is potentially noteworthy that individuals possessing the Tb^1^ mutation resulting in a strikingly short tubby pupa are not readily distinguished from wildtype as adults based on their body length. Given the large numbers of genes impacting human height (Table 1), it is unsurprising that it can be correlated with disparate traits of particular interest (*e.g.*, cancer risk, cardiovascular disease and longevity [Nelson *et al.* 2015; NCD Risk Factor Collaboration (NCD-RisC) 2016)]. Future examination of traits correlated with pupal length (including their reaction norms) may prove informative with respect to their genetic architecture and that of pupal length.

Currently, the automated system does not attempt to distinguish male pupae from female pupae, but it is likely that it could be extended to generate probabilistic sex assignments based on the fact that male pupae are on average ≈8% smaller than females (data not shown). When human height data are corrected for sexual dimorphism [also an 8% average difference between sexes (Galton 1886; Hanley 2004)], there is a corresponding increase of the heritability estimate by 9% (see Table 1 in Hanley (2004)). This implies that analyzing sexed pupal length data will generate heritability estimates that exceed the values reported here for unsexed pupae (Table 2).

The observation that an average 20% ± 11.7 SD of pupae in a vial are not measured by the automated system (Figure 4) is generally not an issue for most experiments (even for select and resequence approaches), as it is often necessary that only a substantial proportion of individuals are measured, which is sufficient to provide a range of parents for the next generation, and to reduce the variance of mean vial estimates (Figure 7). Furthermore, parameters provided in Figure 7 and Figure 8 will provide future researchers with the capacity to readily design maximally resource-efficient experimental strategies.

The observation that the genome scan using 195 RILs (eight-way dataset) failed to identify any significant loci impacting pupal length should be considered in light of the fact that, with this modest number of RILs, the power to detect SNPs of 10 or 5% effect size is only ≈0.37 and ≈0.08, respectively [based on simulations incorporating these exact RILs, see Figure 9 (King *et al.* 2012b)]. Consequently, the results presented here should be viewed as only potentially sufficient to indicate that few, if any, loci of large effect of size are likely to exist for this trait among the RILs used. It is of course conceivable that phenotyping more of the >1600 RILs currently available would identify significant loci (King *et al.* 2012a; Huang *et al.* 2014), and there are several in Figure 10 that are close to the 0.05 significance threshold. Alternatively, an expanding variety of other approaches could be applied *e.g.*, select and resequence (Turner *et al.* 2011; Nuzhdin and Turner 2013; Kofler and Schlötterer 2014), or comparisons between parallel selected lines (Chan *et al.* 2012). While this needs to be further explored in the future, it is clear that the automated phenotyping system will be a key to designing mapping strategies that should allow the identification of many low effect size genes for this trait.

The apparatus used to photograph pupae can be rapidly assembled for ∼500 € (including a camera), or may already be present in many laboratories as geldoc systems. Furthermore the necessary software is open source and free, and runs on any standard desktop PC or MAC. The capacity of the automated system to provide a large number of phenotyped individuals provides increased power to examine the genetic architecture of traits by most approaches. This is likely to prove key to identifying genes or alleles influencing mean pupal length and its variance (Wood *et al.* 2014). This is in addition to potentially facilitating exploring the poorly understood genetic basis of sexual dimorphism (Rawlik *et al.* 2016), and loci which impact the variance of traits [rather than their central values (Ayroles *et al.* 2015)]. In addition, the capacity to select from large numbers of individuals from which to establish future generations has the potential to enhance select and resequence approaches (Turner *et al.* 2011; Nuzhdin and Turner 2013; Kofler and Schlötterer 2014). Furthermore, the large numbers of powerful approaches based on single pair matings developed by animal and plant breeders (Gianola and Rosa 2015) may also become amenable to examine complex traits in *Drosophila* through the use of this simple, low-cost, system. As with human height, over its typical range the genetic architecture of pupal length is of limited practical interest. However, for the last 130 yr, the former continues to provide key insights into the methods through which complex traits can be best understood, this is in part due to the ease and reliability of human height measurement. The high heritability of pupal length, and the capacity to easily automate phenotyping, combined with the small and well-described genome of *D. melanogaster* could make pupal length a similarly valuable model trait.

## Supplementary Material

Supplemental material is available online at www.g3journal.org/lookup/suppl/doi:10.1534/g3.117.039883/-/DC1.

## References

[bib2] Aulchenko Y. S. Struchalin M. V. Belonogova N. M. Axenovich T. I. Weedon M. N., 2009 Predicting human height by Victorian and genomic methods. Eur. J. Hum. Genet. 17: 1070–1075. 19223933 10.1038/ejhg.2009.5 PMC2986552

[bib3] Ayroles J. F. Buchanan S. M. O’Leary C. Skutt-Kakaria K. Grenier J. K., 2015 Behavioral idiosyncrasy reveals genetic control of phenotypic variability. Proc. Natl. Acad. Sci. USA 112: 6706–6711. 25953335 10.1073/pnas.1503830112 PMC4450409

[bib4] Barron A. B., 2000 Anaesthetising *Drosophila* for behavioural studies. J. Insect Physiol. 46: 439–442. 12770207 10.1016/s0022-1910(99)00129-8

[bib5] Barton N. H. Etheridge A. M. Véber A., 2016 The infinitesimal model. bioRxiv 039768.

[bib30] Chan Y. F. Jones F. C. McConnell E. Bryk J. Bünger L. Tautz D., 2012 Parallel Selection Mapping Using Artificially Selected Mice Reveals Body Weight Control Loci. Cur. Biol. 22: 794–800. 10.1016/j.cub.2012.03.011 22445301

[bib6] Galton F., 1886 Regression towards mediocrity in hereditary stature. J. Anthropol. Inst. G. B. Irel. 15: 246–263.

[bib7] Gianola D. Rosa G. J. M., 2015 One hundred years of statistical developments in animal breeding. Annu. Rev. Anim. Biosci. 3: 19–56. 25387231 10.1146/annurev-animal-022114-110733

[bib8] Hanley J. A., 2004 “Transmuting” women into men: Galton’s family data on human stature. Am. Stat. 58: 237–243.

[bib9] Houle D. Mezey J. Galpern P. Carter A., 2003 Automated measurement of *Drosophila* wings. BMC Evol. Biol. 3: 25. 14670094 10.1186/1471-2148-3-25 PMC317280

[bib10] Huang W. Massouras A. Inoue Y. Peiffer J. Ramia M., 2014 Natural variation in genome architecture among 205 *Drosophila melanogaster* genetic reference panel lines. Genome Res. 24: 1193–1208. 24714809 10.1101/gr.171546.113 PMC4079974

[bib11] King E. G. Merkes C. M. McNeil C. L. Hoofer S. R. Sen S., 2012 a Genetic dissection of a model complex trait using the *Drosophila* synthetic population resource. Genome Res. 22: 1558–1566. 22496517 10.1101/gr.134031.111 PMC3409269

[bib12] King E. G. Macdonald S. J. Long A. D., 2012 b Properties and power of the *Drosophila* synthetic population resource for the routine dissection of complex traits. Genetics 191: 935–949. 22505626 10.1534/genetics.112.138537 PMC3389985

[bib13] Kofler R. Schlötterer C., 2014 A guide for the design of evolve and resequencing studies. Mol. Biol. Evol. 31: 474–483. 24214537 10.1093/molbev/mst221 PMC3907048

[bib14] Lamprecht M. R. Sabatini D. M. Carpenter A. E., 2007 CellProfiler: free, versatile software for automated biological image analysis. Biotechniques 42: 71–75. 17269487 10.2144/000112257

[bib15] Mackay T. F. C., 2013 Epistasis and quantitative traits: using model organisms to study gene–gene interactions. Nat. Rev. Genet. 15: 22–33. 24296533 10.1038/nrg3627 PMC3918431

[bib16] Medici V. Vonesch S. C. Fry S. N. Hafen E., 2015 The FlyCatwalk : a high-throughput feature-based sorting system for artificial selection in *Drosophila*. G3 (Bethesda) 5: 317–327. 25556112 10.1534/g3.114.013664 PMC4349086

[bib17] NCD Risk Factor Collaboration (NCD-RisC), 2016 A century of trends in adult human height. Elife 5: e13410. 27458798 10.7554/eLife.13410 PMC4961475

[bib18] Nelson C. P. Hamby S. E. Saleheen D. Hopewell J. C. Zeng L., 2015 Genetically determined height and coronary artery disease. N. Engl. J. Med. 372: 1608–1618. 25853659 10.1056/NEJMoa1404881 PMC4648271

[bib19] Nuzhdin S. V. Turner T. L., 2013 Promises and limitations of hitchhiking mapping. Curr. Opin. Genet. Dev. 23: 694–699. 24239053 10.1016/j.gde.2013.10.002 PMC3872824

[bib20] Polderman T. J. C. Benyamin B. de Leeuw C. A. Sullivan P. F. van Bochoven A., 2015 Meta-analysis of the heritability of human traits based on fifty years of twin studies. Nat. Genet. 47: 702–709. 25985137 10.1038/ng.3285

[bib21] Rawlik K. Canela-Xandri O. Tenesa A., 2016 Evidence for sex-specific genetic architectures across a spectrum of human complex traits. Genome Biol. 17: 166. 27473438 10.1186/s13059-016-1025-x PMC4965887

[bib22] Roff D. A. Mousseau T. A., 1987 Quantitative genetics and fitness lessons from *Drosophila*. Heredity 58: 103–118. 3818341 10.1038/hdy.1987.15

[bib23] Turner T. L. Stewart A. D. Fields A. T. Rice W. R. Tarone A. M., 2011 Population-based resequencing of experimentally evolved populations reveals the genetic basis of body size variation in *Drosophila melanogaster*. PLoS Genet. 7: e1001336. 21437274 10.1371/journal.pgen.1001336 PMC3060078

[bib24] Vinkhuyzen A. A. Wray N. R. Yang J. Goddard M. E. Visscher P. M., 2013 Estimation and partition of heritability in human populations using whole-genome analysis methods. Annu. Rev. Genet. 47: 75–95. 23988118 10.1146/annurev-genet-111212-133258 PMC4037293

[bib25] Visscher P. M. McEvoy B. Yang J., 2010 From Galton to GWAS: quantitative genetics of human height. Genet. Res. 92: 371–379. 10.1017/S0016672310000571 21429269

[bib26] Voss L. D. Bailey B. J. Cumming K. Wilkin T. J. Betts P. R., 1990 The reliability of height measurement (the Wessex growth study). Arch. Dis. Child. 65: 1340–1344. 2270942 10.1136/adc.65.12.1340 PMC1793105

[bib27] Wählby C. Kamentsky L. Liu Z. H. Riklin-Raviv T. Conery A. L., 2012 An image analysis toolbox for high-throughput *C. elegans* assays. Nat. Methods 9: 714–716. 22522656 10.1038/nmeth.1984 PMC3433711

[bib28] Weedon M. N. Lango H. Lindgren C. M. Wallace C. Evans D. M., 2008 Genome-wide association analysis identifies 20 loci that influence adult height. Nat. Genet. 40: 575–583. 18391952 10.1038/ng.121 PMC2681221

[bib29] Wood A. R. Esko T. Yang J. Vedantam S. Pers T. H., 2014 Defining the role of common variation in the genomic and biological architecture of adult human height. Nat. Genet. 46: 1173–1186. 25282103 10.1038/ng.3097 PMC4250049

