# Supplementary material for: Automated Phenotyping Indicates Pupal Size in Drosophila Is a Highly Heritable Trait with an Apparent Polygenic Basis
Source: G3 (Bethesda). 2017 Mar 2;7(4):1277–86. doi: 10.1534/g3.117.039883 (PMC5386876; doi:10.1534/g3.117.039883)
Supplement: Supplementary file 1 [file 1277FigureS1.pdf]

Figure S1 repeat measurements of RILs

A

B

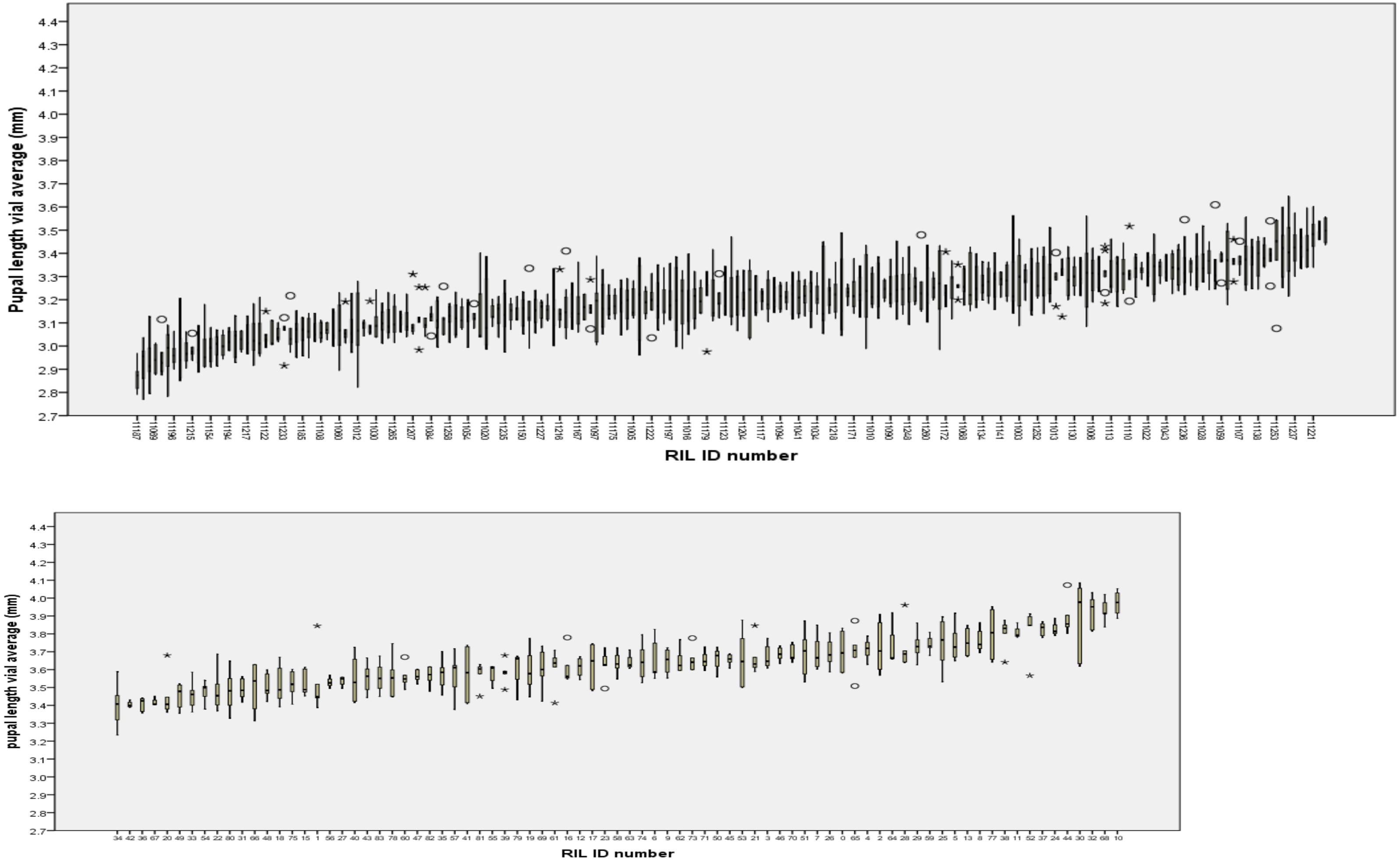


Repeat measurements for each RIL are represented as box plots (A) 8-way dataset, (B) 4-way dataset. Outliers between 1.5-3 times the interquartile range are represented as open circles. Extreme outliers >3 times the interquartile range are represented as stars .The mean number of replicate measurements per RIL in this study was 6.4 ± 2.1SD (5.8% the minimum 4 replicates, 18.0% =5 replicates, 61.2%= 6 replicates and 15% >6 replicates).
