# Supplementary material for: Automated Phenotyping Indicates Pupal Size in Drosophila Is a Highly Heritable Trait with an Apparent Polygenic Basis
Source: G3 (Bethesda). 2017 Mar 2;7(4):1277–86. doi: 10.1534/g3.117.039883 (PMC5386876; doi:10.1534/g3.117.039883)
Supplement: Supplementary file 2 [file 1277FigureS2.pdf]

Figure S2

A

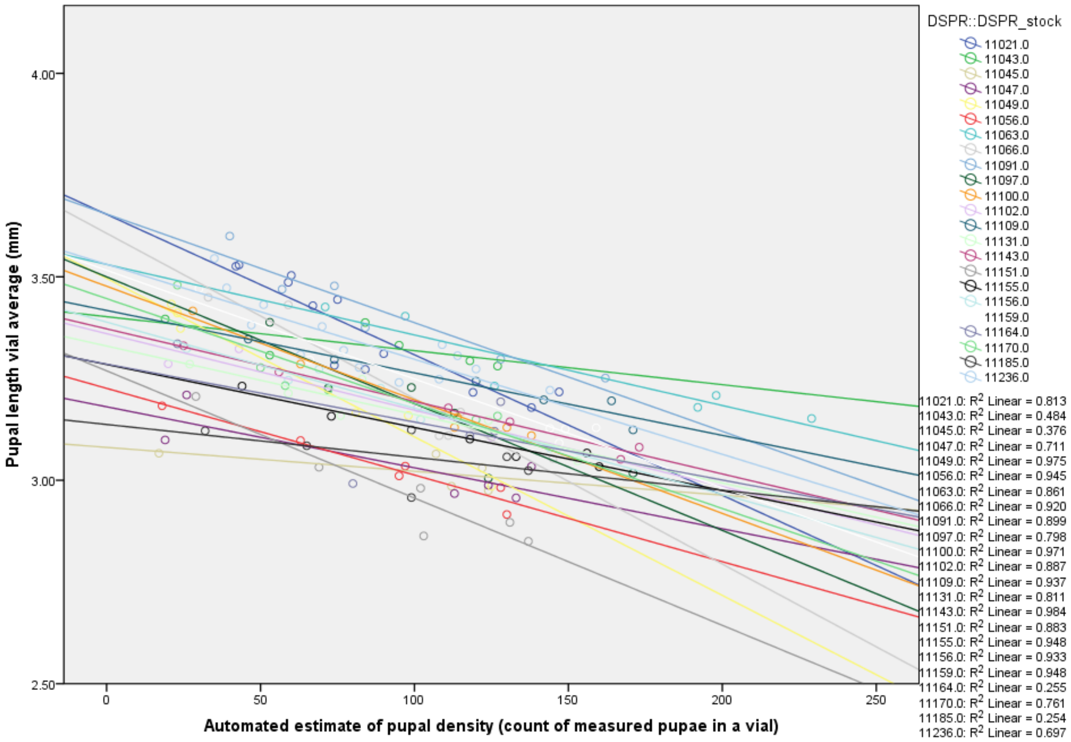

B

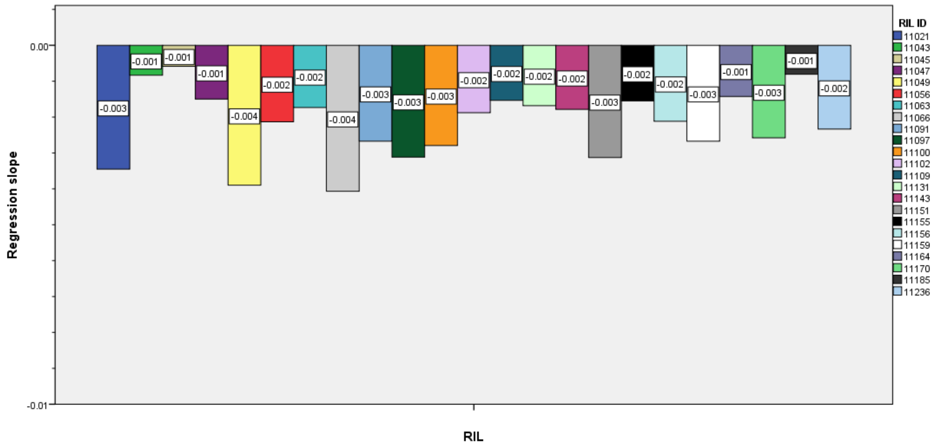

Relationship between vial density and pupal length based on RILs sampled over a wide range of densities.

(A) All RILs (8-way) that were sampled over a range in vial densities greater than 100 were regressed against their mean pupal length estimates. (n=22 RILs). All regressions resulted in negative slopes. (B) The regression slopes are represented for each RIL. The mode slope is -0.002 and is used to correct for density in equation 1. See slope values of -0.0029 and -0.0016 in Figure 5 main text for both the more intensively sampled control lines.
