# Supplementary material for: Automated Phenotyping Indicates Pupal Size in Drosophila Is a Highly Heritable Trait with an Apparent Polygenic Basis
Source: G3 (Bethesda). 2017 Mar 2;7(4):1277–86. doi: 10.1534/g3.117.039883 (PMC5386876; doi:10.1534/g3.117.039883)
Supplement: Supplementary file 3 [file 1277FigureS3.pdf]

Figure S3

A Father only n=431, slope= 0.20, (± 0.026 SE), R^2= 0.117

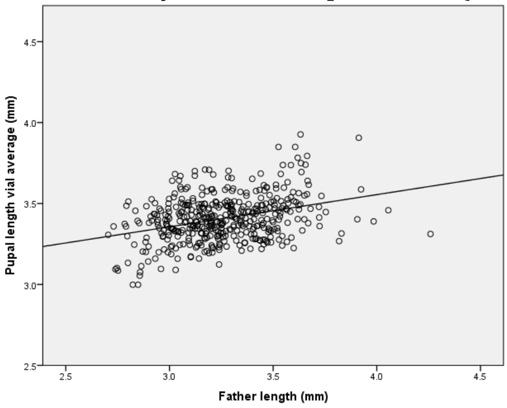

B mother only n=431, slope= 0.12 (± 0.018 SE), R^2= 0.095

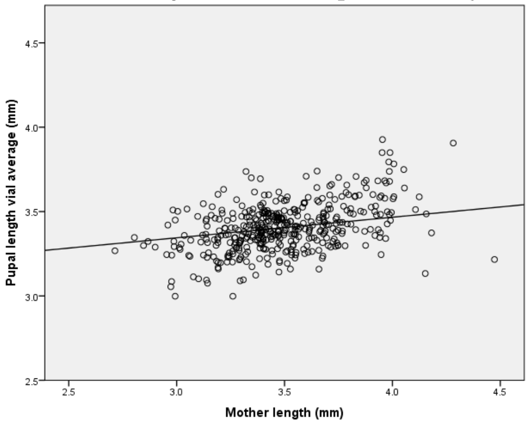

C mid-point parents n=431, slope= 0.44 (± 0.031 SE), R^2= 0.315

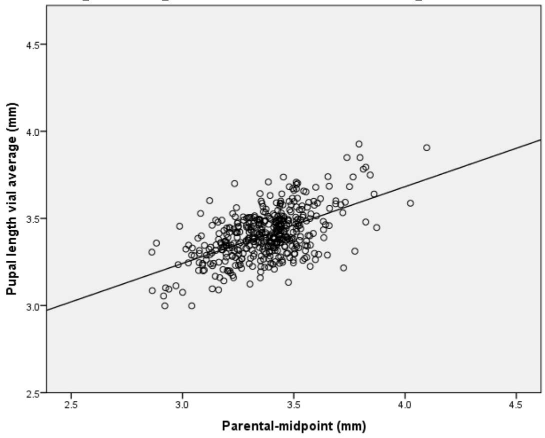

Additive maternal and paternal effects are similar.

The slope of both the maternal and paternal regressions are similar, 0.12 versus 0.20 respectively. For comparison the parental mid-point has a substantially higher slope (0.44) and increased correlation (R^2). This indicates that it is the combined input of both parents rather than a single parent that explains largest proportion of the variance in mean vial pupal length. This parallels observations on Francis Galton's human height data from the 1880s as presented in (Hanley 2004). For the 123 families with ≥4 children the slope values are father =0.38 , mother =0.32, parental midpoint=0.68 (with the respective R^2 values are 0.21, 0.12, 0.31).

Figure S4
