# Supplementary material for: Automated Phenotyping Indicates Pupal Size in Drosophila Is a Highly Heritable Trait with an Apparent Polygenic Basis
Source: G3 (Bethesda). 2017 Mar 2;7(4):1277–86. doi: 10.1534/g3.117.039883 (PMC5386876; doi:10.1534/g3.117.039883)
Supplement: Supplementary file 4 [file 1277FigureS4.pdf]

Figure S4

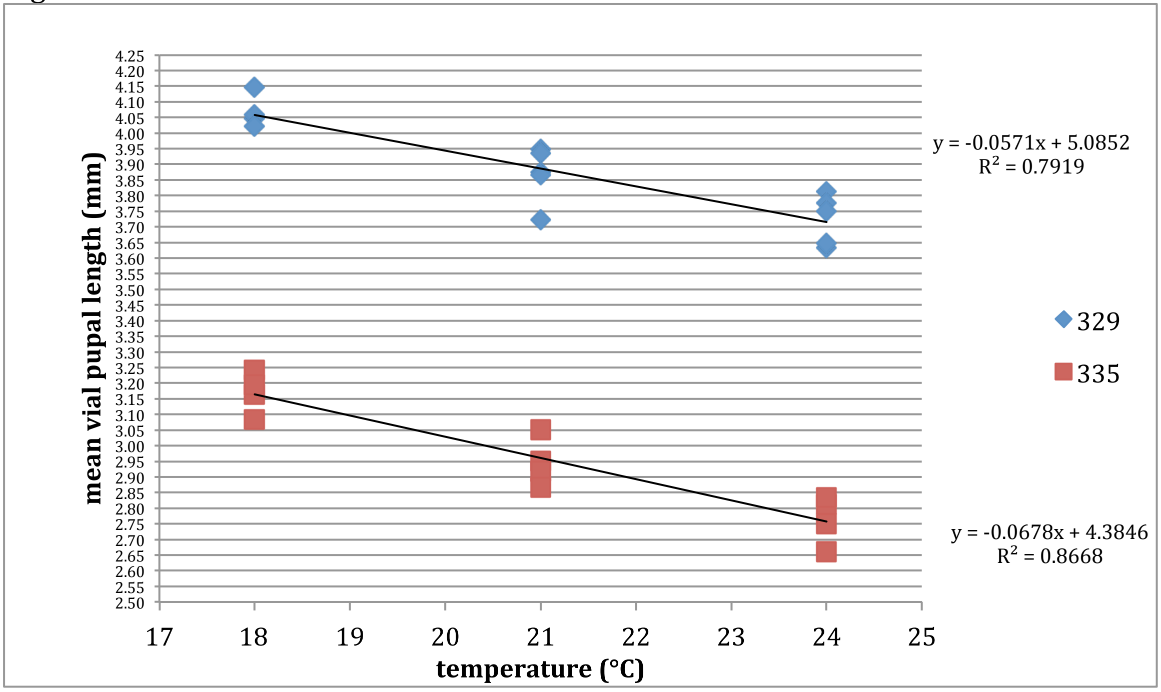

Temperature and its effect on Pupal length.

While the experiments described in the main text were conducted in incubators set to 24° with a 12 hour day night cycle the data for the above experiment were done by submersing the lower 80% of vials in water baths and using natural light. Consequently there may be no direct correspondence between the expectations using the different conditions. The water baths had both heating and cooling units and all data was collected simultaneously in June at our laboratory in Plön, Germany. Groups of adults were introduced into vials with films for 24 hours before being cleared. Resulting films were phenotyped using the automated system. The two control stocks 335 and 329 were used. Four replicates per temperature were measured for each stock.
