# Supplementary material for: Automated Phenotyping Indicates Pupal Size in Drosophila Is a Highly Heritable Trait with an Apparent Polygenic Basis
Source: G3 (Bethesda). 2017 Mar 2;7(4):1277–86. doi: 10.1534/g3.117.039883 (PMC5386876; doi:10.1534/g3.117.039883)
Supplement: Supplementary file 5 [file 1277FigureS5.pdf]

Figure S5

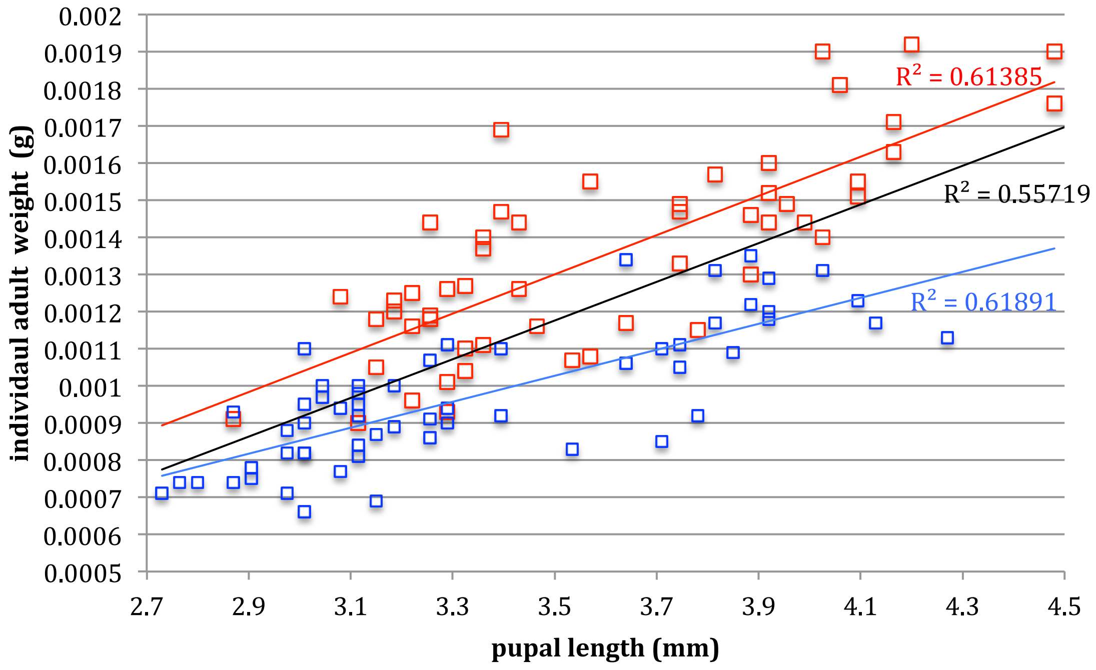

Correlation between pupal length and adult weight.
60 male and 60 female pupae from different vials across the entire sampled range of lengths and were weighed as 1 day old adults (wet weight). Red points =females, blue =males. Sex specific correlations (n=60) are shown in color and for unsexed individuals (n=120) in black.
