# Supplementary material for: Automated Phenotyping Indicates Pupal Size in Drosophila Is a Highly Heritable Trait with an Apparent Polygenic Basis
Source: G3 (Bethesda). 2017 Mar 2;7(4):1277–86. doi: 10.1534/g3.117.039883 (PMC5386876; doi:10.1534/g3.117.039883)
Supplement: Supplementary file 6 [file 1277TableS1.pdf]

Table S1 Information on founding stock of RILs

Founder 8-way cross

Founder	DSPR Vial Code	Original Stock Center	Original Stock Center Stock Number	Stock Name	Available vial average pupal length[1]
A1	b1	Bloomington	1	Canton-S	3.2mm±0.1 SD(n=3)
A2	b3841	Bloomington	3841	BOG1	3.0mm±0.1 SD(n=3)
A3	b3844	Bloomington	3844	BS1	3.3mm±0.1 SD(n=3)
A4	b3852	Bloomington	3852	KSA2	3.5mm±0.2 SD(n=3)
A5	b3875	Bloomington	3875	VAG1	3.3mm±0.1 SD(n=3)
A6	b3886	Bloomington	3886	wild5B	3.1mm±0.1 SD(n=3)
A7	T.7	Tucson	14021-0231.7	n/a	3.0mm (n=1)
AB8	Sam	TFC Mackay	n/a	Sam; ry506	3.3mm±0.1 SD(n=3)

[1] not all vials had ≥15 measured pupae, unlike in main datasets.

The establishment of the RILs is described in (King et al. 2012) as follows

'In the first generation, lines were intercrossed (e.g., A1 3 A2, A2 3 A3, . . . AB8 3 A1), and 10 F_1 flies per genotype per sex were used to establish the next generation. Adult F_2 flies were used to a 1/2 gallon population bottles, [..] maintained independently by transferring adults en masse to fresh bottles every 12–13 d.'

Inbreeding of lines occurred after G50. For a list of all 195 RILs phenotyped see File S6.

Founders 4-way cross

Stock name	Stock Center	Original Stock Center Stock Number	Collection location	Available vial average pupal length[1]
329	EHIME (Watada)	TS48	Fukuoka, Kyusyu, .Japan	3.74mm±0.1 SD(n=162)
330	EHIME (Watada)	TS95	Fukuoka, Kyusyu, .Japan	3.56mm±0.12 SD(n=47)
335	EHIME	ZI178-Davis	Siavonga, Zambia	2.98mm±0.08 SD(n=98)
340	EHIME	UM6-Davis	Masindi, Uganda	3.01mm±0.08 SD(n=43)

All 4 stocks were checked microscopically (n=>5 individuals) for departures from the standard karyotype, none were found.

The 4 way cross was generated in the following manner using only single pair crosses.

G0	[329 female x 340 male]**a** and [330 female x 335 male]**b**
G1	single cross **a** male x b **b** female
G2	15 single pair crosses between randomly selected siblings.
G3	81 single pair crosses between randomly selected individuals from different vials
G4	78 single pair crosses between randomly selected individuals from different vials
G5	88 single pair crosses between randomly selected individuals from different vials
G6	154 single pair crosses between randomly selected individuals from different vials
RIL	G15 –G35 inbreeding sib-matings to generate 81 RILs.
