# Supplementary material for: Automated Phenotyping Indicates Pupal Size in Drosophila Is a Highly Heritable Trait with an Apparent Polygenic Basis
Source: G3 (Bethesda). 2017 Mar 2;7(4):1277–86. doi: 10.1534/g3.117.039883 (PMC5386876; doi:10.1534/g3.117.039883)
Supplement: Supplementary file 7 [file 1277FileS1.docx]

File S1 Detailed description of imaging system.

*Vial preparation.* Food using the following recipe was prepared; 8g agar, 108g corn/soy flour mix, 80g malt extract syrup, 22g sugar beet syrup+ water to a total weight of 1234g +6.25ml propionic acid +10ml Nipagin. This was dispensed into standard 28.5mm diameter, 95mm height vials (Genesee Scientific), using a ‘*droso-filler’* (Genesee Scientific) to a depth of approximately 15mm. At any point after the food had set 10 x10.5cm squares of transparent film were slid all the way down to the bottom of the vial, long edge down. The film protrudes 1 cm above the top of the 9 cm high vials to make it easier to grasp the film for removal (see figure below). The film is designed for use in old-style overhead projectors and is still widely available, we used plain paper copier film, (nobo, 33638237). While this type of film has a rough and smooth side we found no obvious difference between the two and in practice ignored it. Using a guillotine it is possible to cut 600 sheets at a time into squares of the specified size (6 squares per sheet), at a cost of ≈0.4 cent each. Inserting the film does not appear to change the storage properties of the food. Prior to introducing adults a very small amount of live yeast paste was dotted on the surface using a disposable pipette tip.


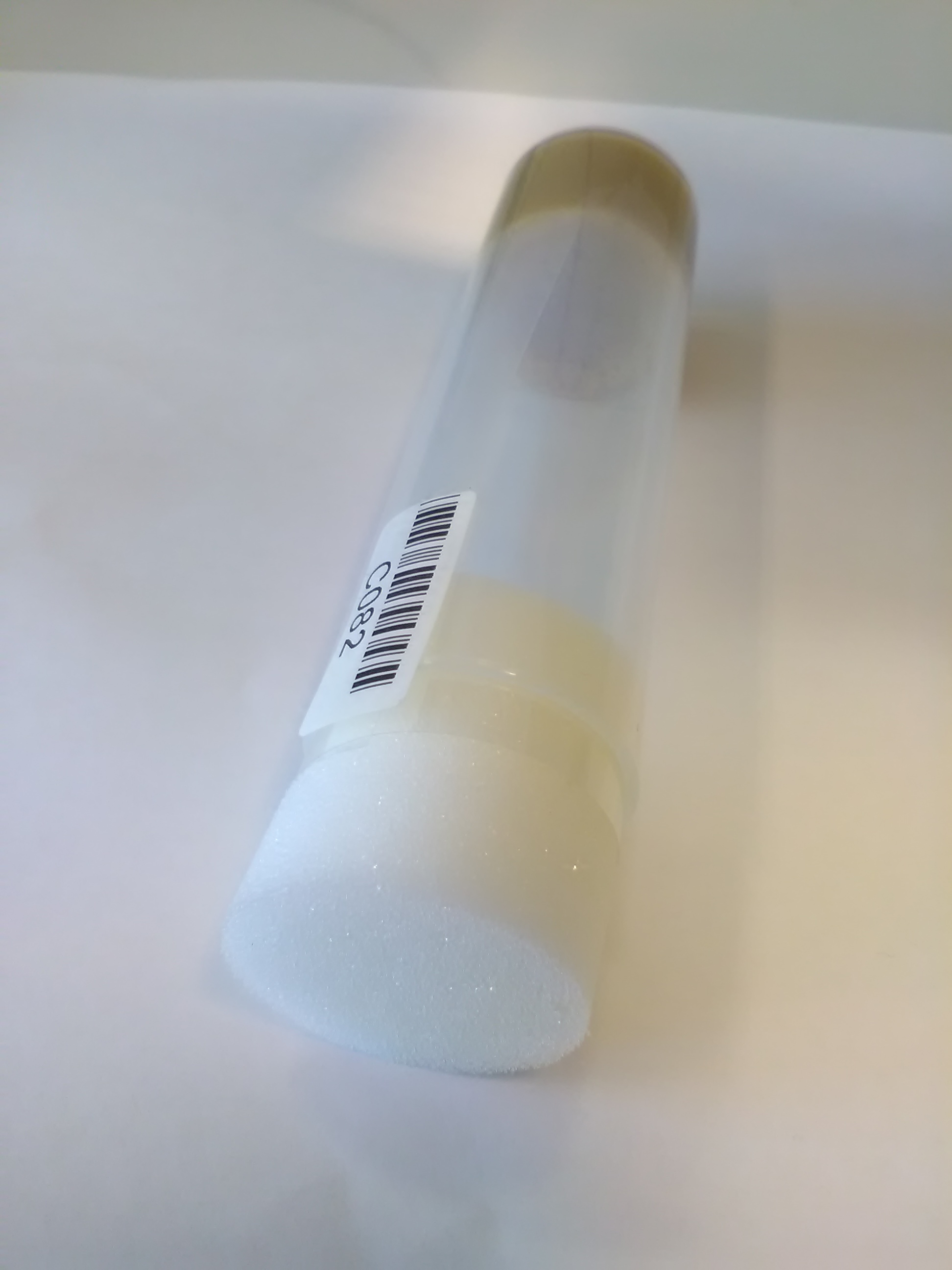

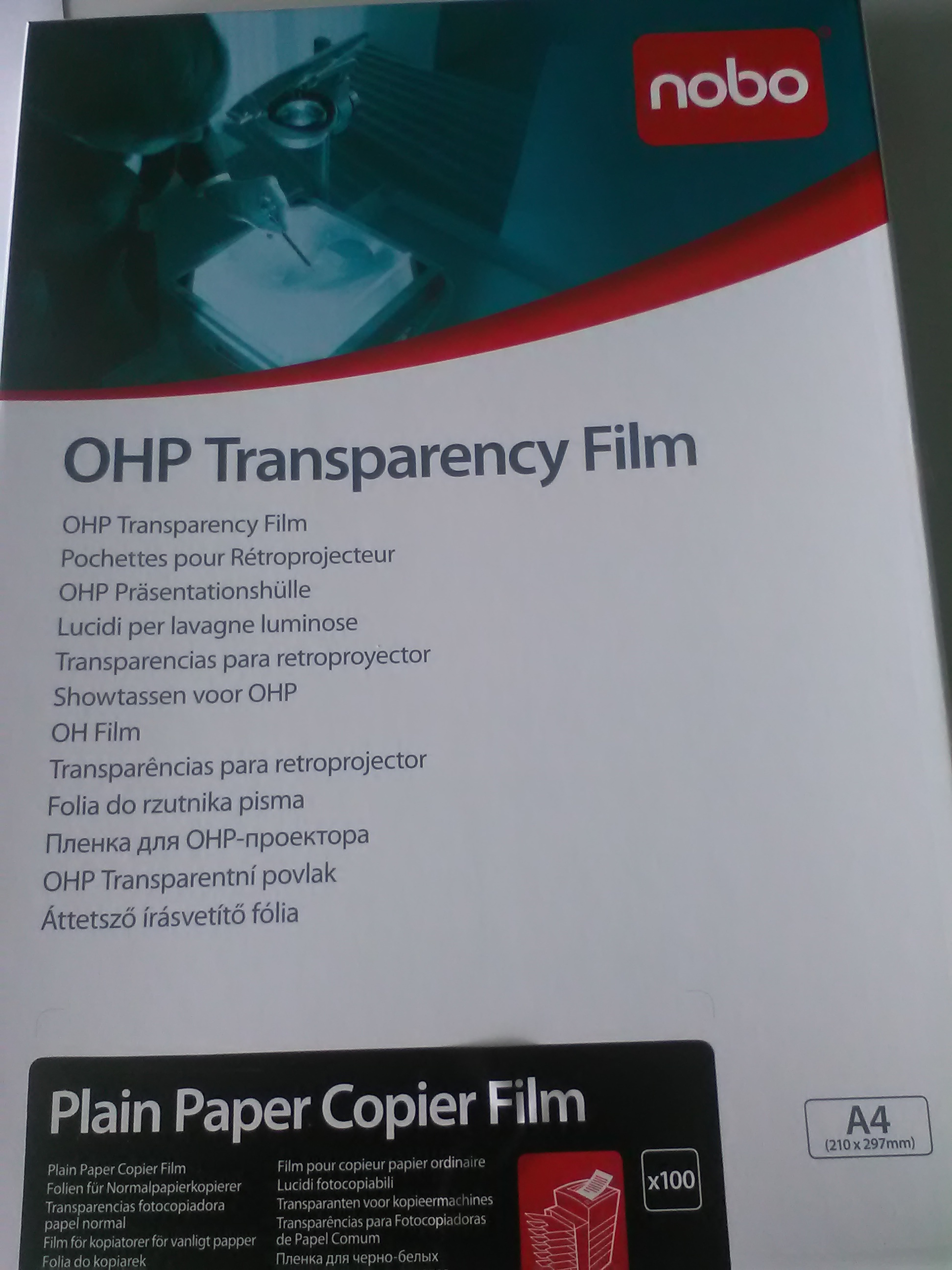


**Right,** Vial with the transparent film in place. The film visibly extends beyond the upper edge of the vial even when pushed down to the bottom of the vial. The semi-transparent barcode sticker that is removed from the vial and placed on the film upon its removal is also visible. **Left,** Brand of Overhead Projector Film used, which is still widely available.

*Single pair matings* were established using sexed adults that had not been anesthetized and were 1-3 days old (see below). While it was not explicitly tested the low failure rate of single pair matings, ≈4 % for outbred parents and ≈10% using RILs, may be due to avoiding C0_2_ exposure (%s based on a subset 401 vials and 77 vials respectively). Exposure to C0_2_ is known to delay copulation in (Barron 2000) addition to being near lethal to individuals exposed prior to wing expansion (see figure 8.53 in ((Ashburner *et al.* 2011)). Adults were generally placed in vials with films for 2 days (occasionally up to 4 days if a low egg laying rate was noticed or due to weekends).

The relatively short time adults spend in vials helps to ensure that the majority or all individuals have synchronously developed to pupae by the time the film is removed. This improves the extent to which the density of vials can most meaningfully be estimated from automated pupal counts (which could be misleading if a substantial proportion of larvae remain in the food after film removal). Furthermore, the prompt removal of adults increases the probability that both parents can be recovered and stored for retrospective molecular analysis.

*Matings of groups of individuals from an RIL*. Individuals 2-5 days old were briefly anesthetized using CO_2_ and separated into groups of 12-16 and introduced into vials with films. Adults were cleared after one night, either thrown away or into an additional vial where replicate vials were required.

*Control measurements.* Throughout the experiment two stocks were continually re-measured in the manner described above for RILs to control for environmental effects. These were a long stock (329) and a short stock (335)- see Table S1. The former stock had a consistently higher fertility than the latter consequently groups of 5-8 or 12-16 individuals were used for stock 329 and 12-16 for stock 335.

During the experiment period covered by the study the main incubator used for experiments was replaced at week 90 in addition to a change in the height of the camera. The change in the height of the camera was corrected using the diameter of the coin that is part of all images. Though both incubators had been set to be 24° there was a 4% change in the average length of the control pupae (which was not connected to the camera height). This was probably due to the fact that the earlier incubator spent the 12 hours of darkness at 22.7° ±0.19SD and the 12 hours of light at 24.1° ±0.25SD while the corresponding values for the new incubator were 24.1° ±0.04SD and 24.0° ±0.03SD (measures were taken continuously using a Hobo U12-012 data logger, ONSET)


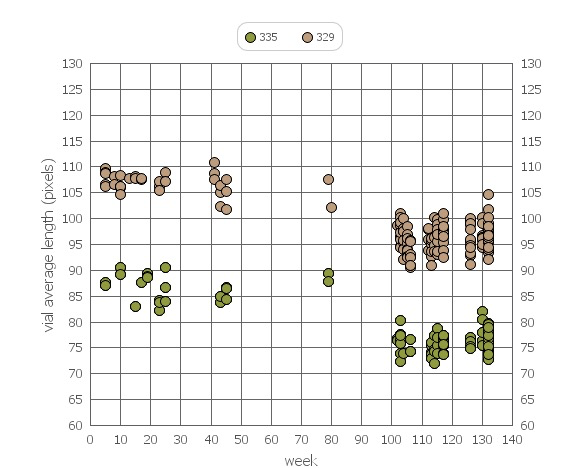


**Raw replicate measurements of the two control stocks throughout experiments (pixels)** At week 90 an incubator was replaced and also the camera height changed.


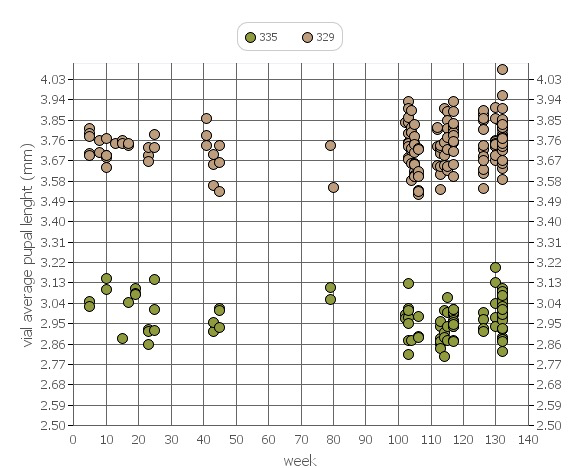


**Adjusted replicate measurements of the two control stocks throughout experiment (mm).** After week 90 all measurements (including experimental ones) were increased by 4% to allow for the impact of changing an incubator.

*Photography of films.* Films were removed from vials when most or ideally all larvae had attached to the film as pupae. This was generally around the 10^th^ day after the vial was initiated, often when some of the earliest pupae had become their darkest prior to eclosion (a small number of pupae may actually have eclosed on some films). Monitoring the vials from which films had been removed confirmed that generally the majority had few or no larvae that subsequently developed to pupae.


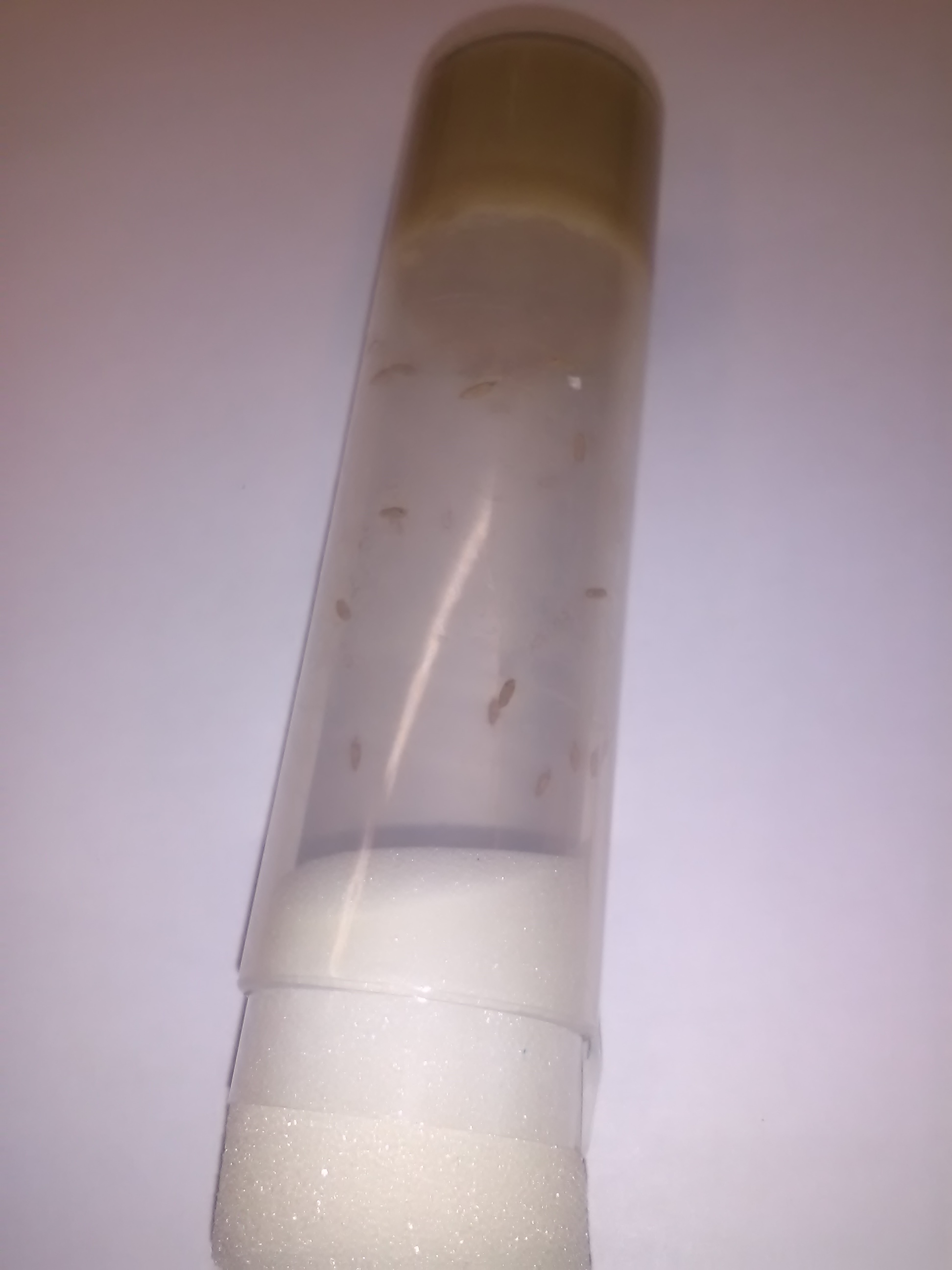

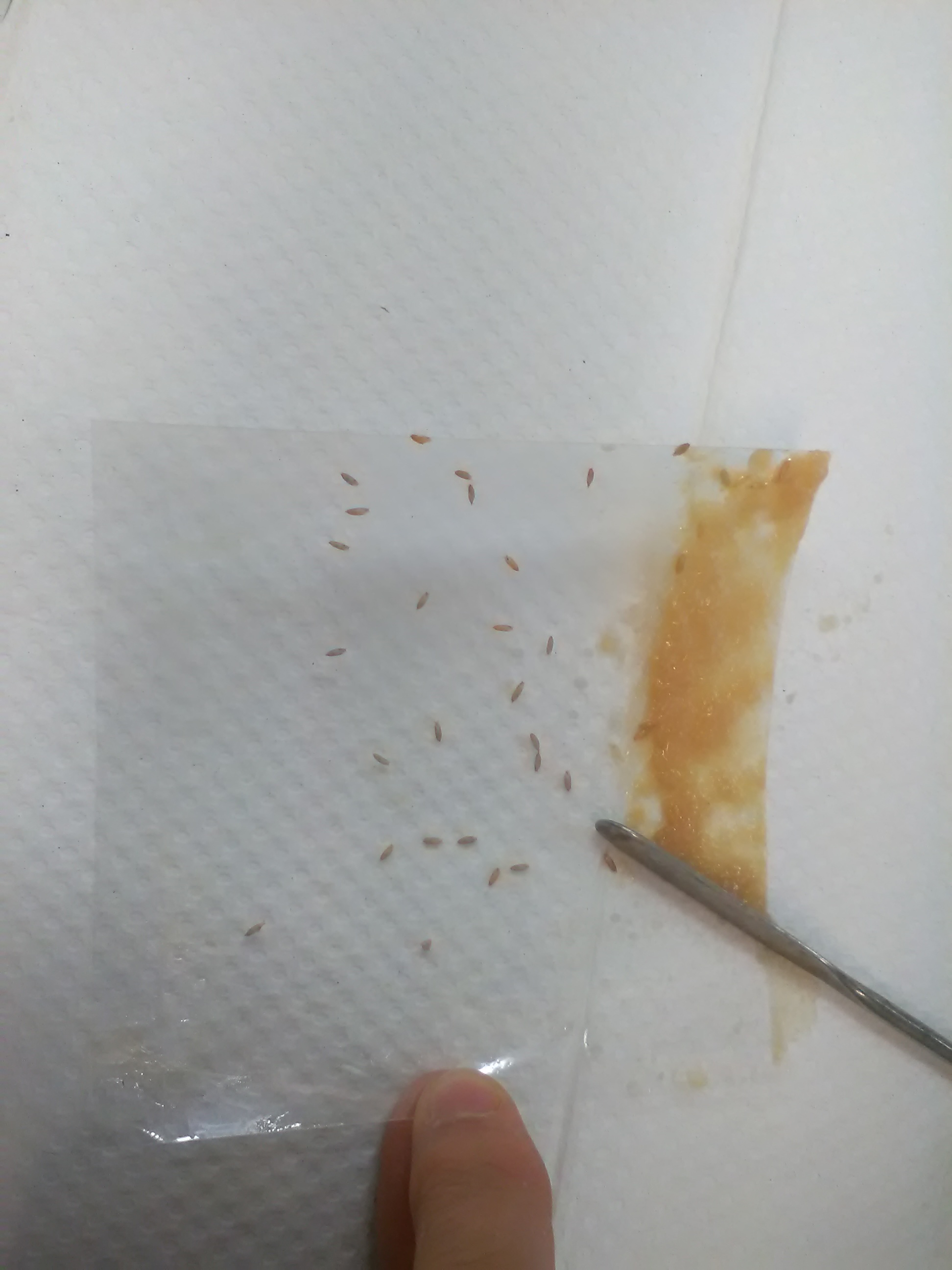

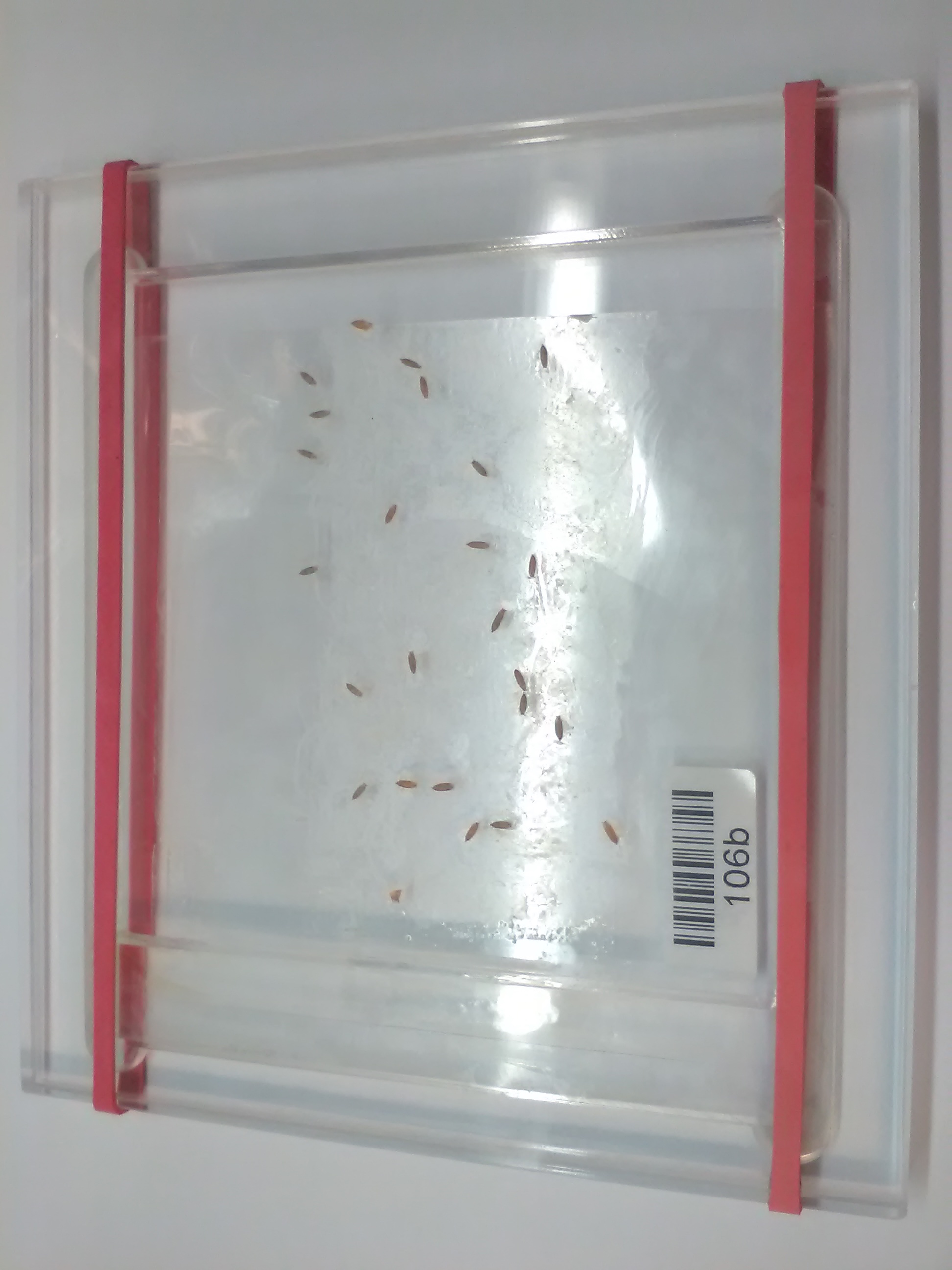


**Left**, Vial with pupae. **Center**, removing pupae obscured by food. **Right**, film placed in frame including semi-transparent label. The read elastic bands used to keep the frame and its transparent back-plate are clearly visible.

Any food or pupae whose edges were obscured by food were scraped away from films using a metal spatula (see Figure above). Generally only a small number of pupae needed to be removed in this manner as most pupae attach >>5mm above the food surface. Films were placed individually in a custom frame that held them flat and which permitted illumination from below. The film was held in place by a 2mm thick back plate of transparent plastic, this was retained by two rubber bands (see above) Files for 3D printing are provided as File S2, which can be printed using any plastic and paired with squares of 2mm transparent plastic. Once the film was in place the sticky label barcode was removed from the vial and placed on the film in the lower left corner. Any crawling pupae were removed using tweezers, and films in frames were then left in a 24° incubator for >1hour to ensure that all pupae had developed beyond stage P2 (i.e. no longer white puparium stage P1)- though this step was generally unnecessary for most films. Frames were then photographed in a light tight box with a sliding door, which provided illumination only from underneath the frame, effectively silhouetting the pupae while minimizing tangential shadows. Illumination was provided from a uniform intensity white A5 electroluminescent light sheet (approximately 30€ posterpoweruk.co.uk or equivalent from glowhut.com).


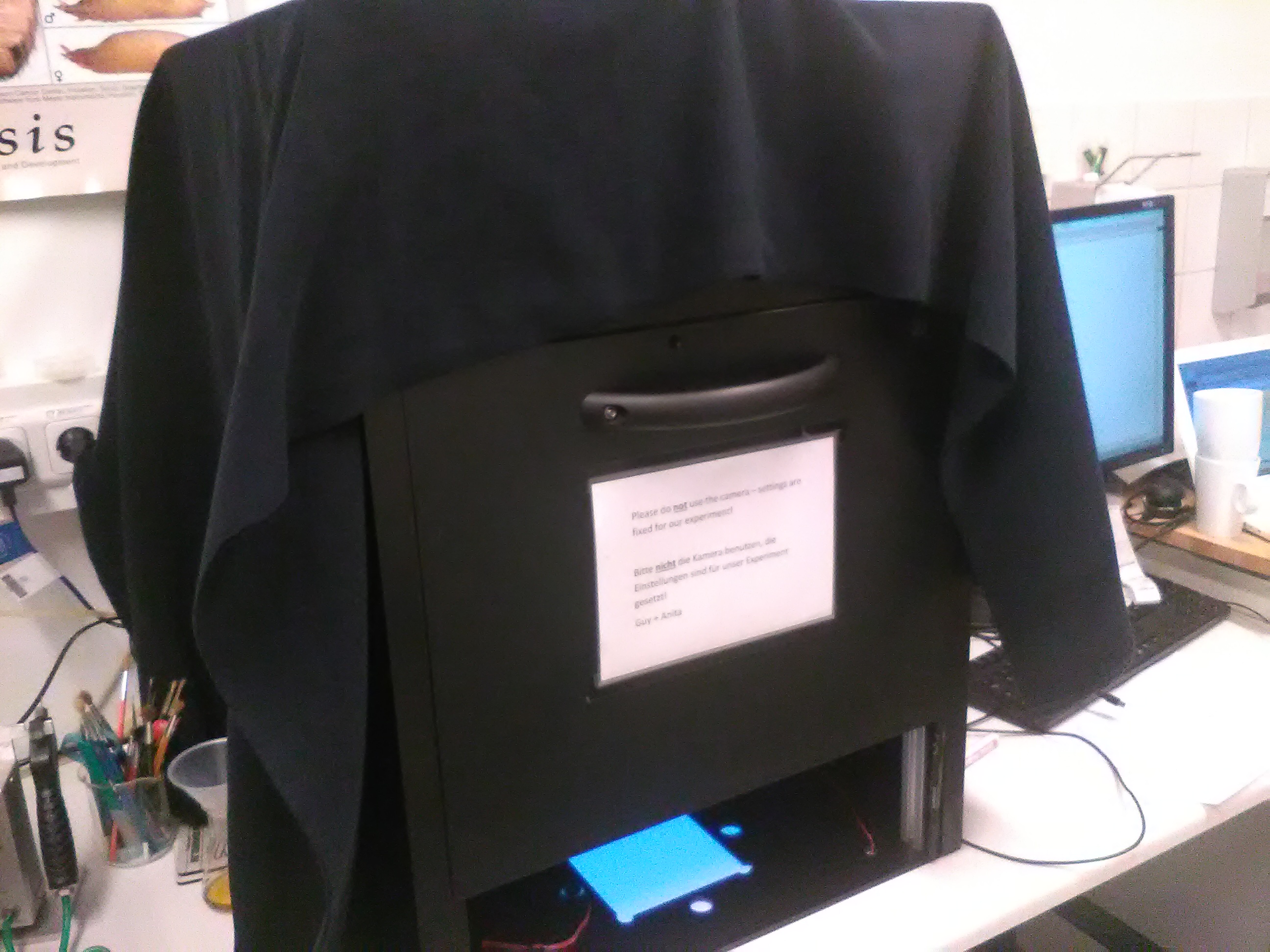

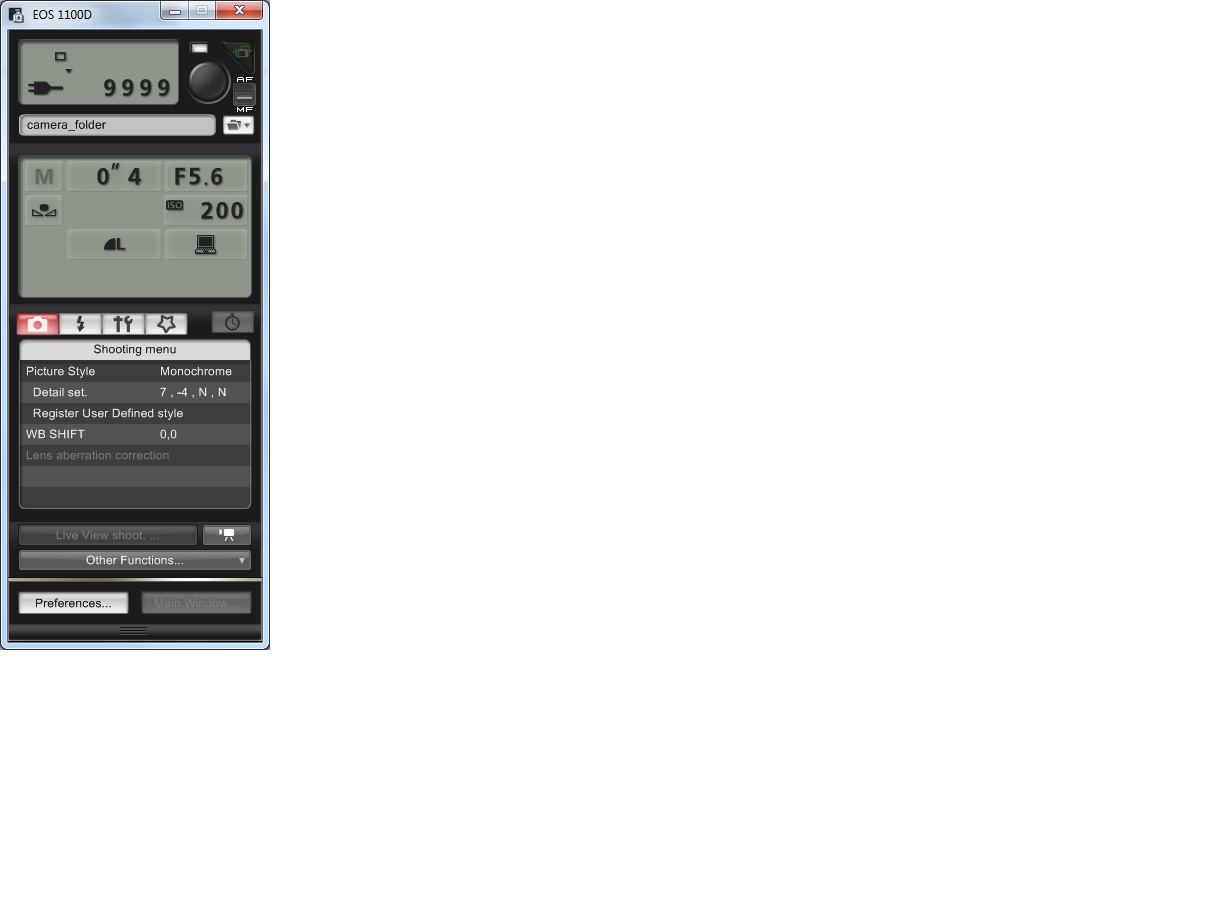


**Left**, The camera is positioned at the top of the box and controlled by the adjacent computer. The door was slid down for each picture. The light sheet which provides the illumination is visible in the area which holds the frames. A black bit of cloth covered the top of the box to provide access to the camera when necessary. **Right**, camera settings for the Canon EOS 1100D.

The light sheet was left on for more than 30 minutes prior to photographing as there was an up to 12% change in intensity over this period (though this did not result in any change of measurements) . The camera was powered through a mains supply and controlled entirely through a connected PC interface. The camera was set to a fixed manual exposure of F=5.6 and ISO=200.The 18-55 mm zoom camera lens was approximately 27cm from the film and set to take monochrome images. The 16.25mm diameter coin had a diameter of 434pixles (if you photos are within 7% of this scale, and there is a high contrast between pupae and background then the Cellprofiler pipeline in File S3 should work without modification)

The camera focus was set from the computer at the beginning of each photography session. Photographs of a ‘test film’ with numerous marker pen lines representing pupae was used for focusing and to confirm the stability of all settings. Files (.jpg) where named automatically by the camera using a sequential number and the current date. Files were approximately 2.5-3Mb in size. Every photograph included a 1cent € coin which had been sprayed black. This ensured that all measurements in pixels could be converted to mm. To enable the barcode stickers to be automatically read from the image taken using only bottom illumination it is necessary for the stickers to be semi-transparent and on a white background (it will not work reliably in our experience using standard opaque stickers or fully transparent ones). This was achieved using a custom label (HBCL printed with two layers of white, 1’’ x 0.4375’’, A00666-02189 GA International Inc., cost ≈ 55€ per roll of 1000) and printed using a thermal transfer printer (Zebra GX430T). Stickers included human and machine-readable (128-code) versions of a unique identifier intended to be the primary-key in the database and form the archived filename of the image.


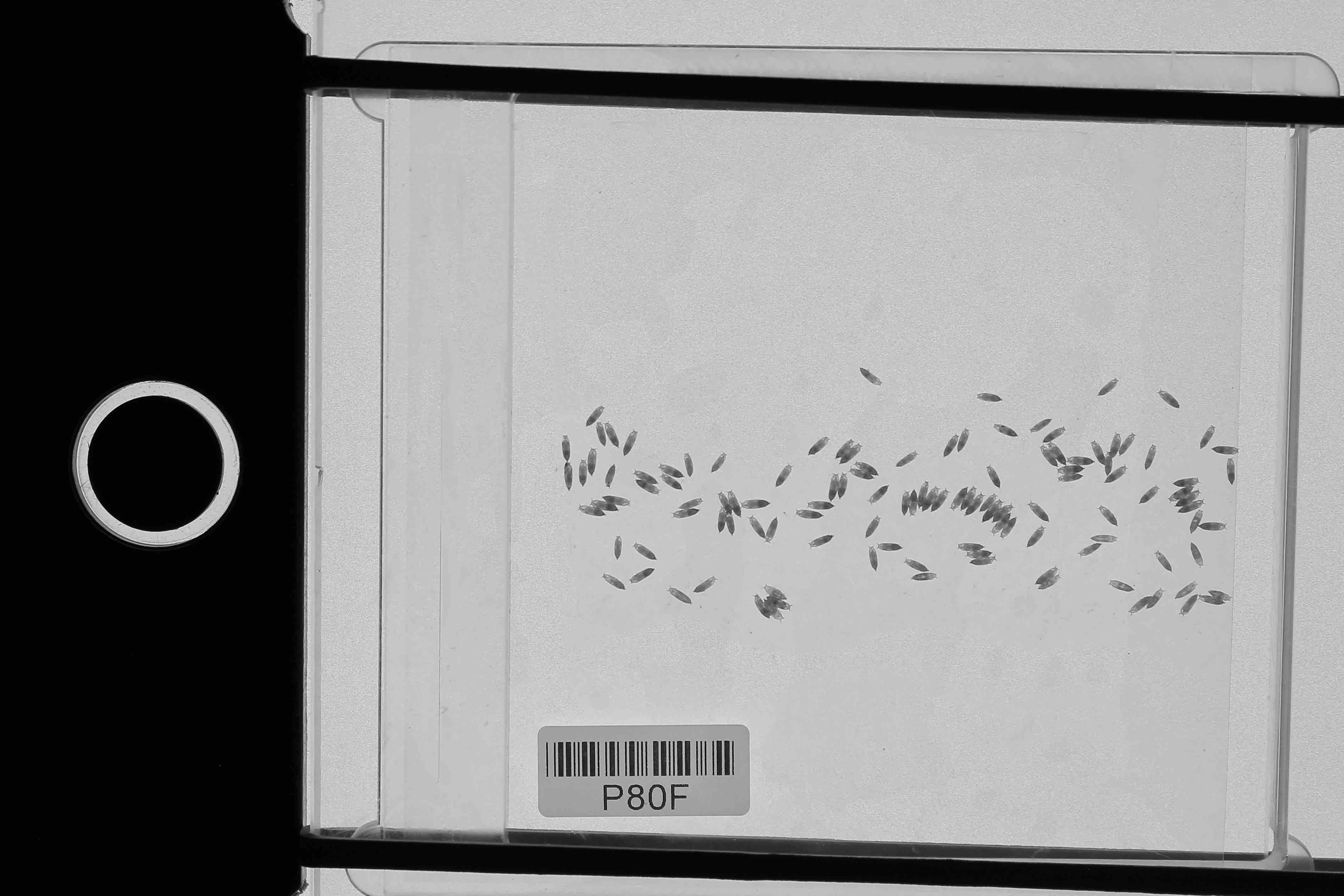


Typical image. Note the black 1€ cent coin on the left and is 16.25mm in diameter. Note the high contrast between pupae and background.

*Analysis of .jpg files.* While not strictly necessary, to assist with archiving, folders of .jpg files were imported into Filemaker where the barcodes were automatically read from images and used to rename the file with a custom script. These renamed .jpg files were then imported into Cellprofiler. Each module in the pipeline is annotated to describe its purpose and highlight key parameters that could be useful to change for different setups (File S3). Two additional files are necessary to run the pipeline. The first is a model of pupae shape used by the ‘UntangleWorms’ module. The second is a ‘seed pixel’ that must be located within the area of the image where the coin is, this is used by the pipeline to quickly identify the location of the coin without having to search the entire image for it (module:IdentiySecondaryobjects- the first use in the pipeline). Both modules need to be provided with the location of the respective files. We have found that the pipeline runs successfully with little or no adjustment over a wide range of camera settings. Though the pipeline is most successful when the contrast between pupae and the background is high. There are several modules in the Cellprofiler pipeline where objects either too small or too big to plausibly be pupae are filtered out, consequently it is best the camera is set so pupae are within the range 60-130 pixels in length. Optimal camera height can easily be found using Cellprofiler File>View image>Tools>Measure length. Using a desktop PC each image was processed in approximately 3 minutes. The output files of the pipeline is as follows (see ‘Output’ folder File S3).

For each .jpg

Area of original image where pupae where searched for- in ‘crop’ folder

Outlines of numbered objects overlaid on original image (image made lighter to make it easier to read numbers)- in ‘all_outlines’ folder. See also Figure 2 of main text

For each batch of .jpgs analyzed input in a common folder multiple .cvs files are generated, the most useful are listed below with key parameters described. Other files and measurements are present and are described in the Cellprofiler manual.

*Image.csv* describes general features of each image, of limited general use but can be valuable in troubleshooting thresholds when setting up camera.

*coinSECONDARY.csv*  Provides various measurements of the coin. ‘AreaShape_MajorAxisLength’ colum is used to convert pixels into mm.

*pupaeSECONDARYobjects.csv* Provides various measurements of each pupae. The following fields are key ones.

‘Metadata_barcode’ is the input file name without extensions.

‘ObjectNumber’ is the number overlaid on image.

‘AreaShape_MajorAxisLength’ used as pupal length measurements (units are pixles). Length is formally defined in the Cellprofiler manual as ‘*The length (in pixels) of the major axis of the ellipse that has the same normalized second central moments as the region.* ’

High confidence pupae

Children_pupaeSECONDARYobjectsFILTEREDhigh_Count =1

Children_pupaeSECONDARYobjectsFILTEREDlower_Count=1

Medium confidence pupae

Children_pupaeSECONDARYobjectsFILTEREDhigh_Count =0

Children_pupaeSECONDARYobjectsFILTEREDlower_Count=1

Low quality objects

Children_pupaeSECONDARYobjectsFILTEREDhigh_Count =0

Children_pupaeSECONDARYobjectsFILTEREDlower_Count=0

All outline.jpg files with their associated ‘*pupaeSECONDARYobjects.csv*’ and ‘*coinSECONDARY.csv*’ were imported into the database. Within the database aberrantly measured pupae could easily be excluded from analysis.

A fully annotated version of Cellprofiler pipeline, setup instruction, an example test file and expected output files are all provided in File S3.

*Full list if measurements reported by Cellprofiler pipeline.*

Morphological measurements

AreaShape_Area

AreaShape_Compactness

AreaShape_Eccentricity

AreaShape_EulerNumber

AreaShape_Extent

AreaShape_FormFactor

AreaShape_MajorAxisLength

AreaShape_MaxFeretDiameter

AreaShape_MaximumRadius

AreaShape_MeanRadius

AreaShape_MedianRadius

AreaShape_MinFeretDiameter

AreaShape_MinorAxisLength

AreaShape_Perimeter

AreaShape_Solidity

Position and orientation

AreaShape_Center_X

AreaShape_Center_Y

AreaShape_Orientation

Location_Center_X

Location_Center_Y

Reporting of nearest neighbors

Neighbors_AngleBetweenNeighbors

Neighbors_FirstClosestDistance

Neighbors_FirstClosestObjectNumber

Neighbors_NumberOfNeighbors

Neighbors_PercentTouching

Neighbors_SecondClosestDistance

Neighbors_SecondClosestObjectNumber

Various intensity measurements are also reported by the Cellprofiler pipeline that broadly equate to the color of the pupae (see expected results folder File S3 ‘pupaeSECONDARYobjects.csv’). These can be useful if attempting to automatically identify eclosed pupae.

The majority of the study is based on ‘AreaShape_MajorAxisLength’ that is defined as

the length (in pixels) of the major axis of the ellipse that has the same normalized second central moments as the region of the object. The definitions of all the above measurements are detailed in the Cellprofiler manual.

E*xclusion of aberrant pupae*

The Cellprofiler pipeline is intended to outline all identified objects that are likely to be pupae. Objects which do not conform to the following criterion are filtered out by the final ‘Filter Objects’ modules:-

Medium quality objects

AreaShape_MajorAxisLength min 60 pixels max 135 pixels (2.3-5.1mm)

AreaShape_MinorAxisLength min 19 pixels max 42 pixels (0.7-1.6mm)

High quality objects

AreaShape_MajorAxisLength min 60 pixels max 135 pixels (2.3-5.1mm)

AreaShape_MinorAxisLength min 19 pixels max 42 pixels (0.7-1.6mm)

Intensity_UpperQuartileIntensity 0.5-0.9

Neighbors_NumberOfNeighbors=0 (within scale of 4 pixels)

However good an automated system there is always likely to be a number of errors. Generally for most analyses it is not necessary that all pupae are measured, only that a substantial proportion are reliably measured.

A small proportion of pupae are completely missed by the automated system (Figure 4). However, assuming that the probability of being missed is random with respects to pupal length this will have minimal impact on the estimation of vial means and variance. Pupae which have been measured incorrectly have a greater capacity than missed pupae to influence the estimation of vial means and variance. Within the Filemaker database it is possible with a single click to manually exclude from analysis putative pupae identified by the automated system that do not conform to the normal shape of pupae.

Examples of canonical pupae and aberrant pupae are shown below (magnification is not uniform).

Examples of canonical pupae used in length analysis


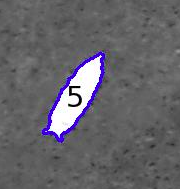

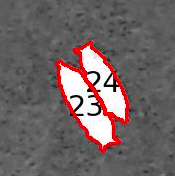

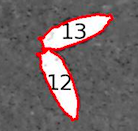

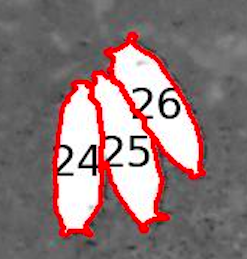


Examples of aberrant pupae manually excluded from length analysis.


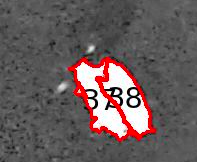

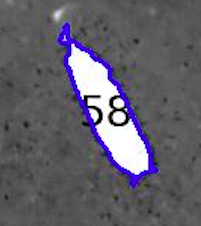

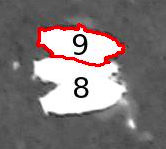


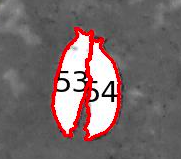

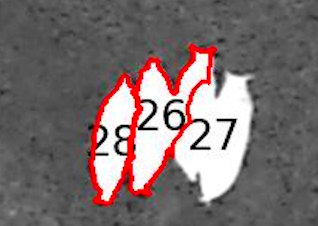


Multiple factors can be simultaneously involved in causing aberrant measurements and include.

Dirt or food near pupae.

Pupae with close neighbors are inherently more difficult for the automated system to resolve.

Very occasionally dead larvae were present in the pictures.

The probability of pupae being manually excluded as aberrantly measured was 0.02 for high quality pupae and 0.10 for medium confidence pupae (blue and red outlines respectively).

*Selecting phenotyped pupae for crosses in subsequent generations.* The capacity to select phenotyped individuals for subsequent crosses is essential for generating trios for h^2^ estimates or in selective breeding strategies. While waiting for the Cellprofiler pipeline to finish analyzing and the results imported into the database, films were kept in humid plastic containers. Films in frames could be kept overnight in this way, though some individuals may eclose and be effectively lost. Where convenient films could be placed in a 9-12° fridge (the same used to hold prepared food) for 2 or 4 days without obvious loss of viability.

Using the Cellprofiler overlaid image were each pupae is numbered it is possible to identify phenotyped individuals. Were any ambiguity exists it is possible to hold the frame up to a 1:1 image either on a screen or tablet. To setup single pair crosses it is necessary to sex pupae. This was routinely done in one of two ways.

First if the pupae were dark (stage P12-P15) then by inverting the plate it is straightforward to determine if sex combs are present on the front legs (only present in males) using a standard dissecting microscope or magnifying glass. With a wet paintbrush, used for standard fly pushing, it is possible to lift individual pupae from the film while leaving adjacent ones undisturbed. If this proves difficult then misting with water the whole film using a domestic spray bottle and leaving for a minute will resolve this. In this manner pairs of male and female pupae can be selected and placed in new vials with films to form the next generation. To keep pairs of selected pupae from resting on the new food surface parafilm or the sticky part of a post-it-note can also be used to place the pupae on.

The second technique was used routinely as it can be used at any pupal stage past P1 (P2-P15). Individual pupae were removed from films using brushes as described above, these were then placed separately into cuvettes. These cuvettes were reused for this purpose multiple times. On the outside part of the cuvette lid was a unique barcode sticker that enabled the phenotype and ancestry data of the pupae within each cuvette to be related through the database. A small disk of paper soaked in adult food was placed in the lid of the cuvette to provide food to the eclosed individual. The disk was made by using a ring binder hole punch on filter paper. The adult food disks were soaked in a solution approximately comprised; 30 % water, 60% red grape juice and 10% dried yeast. Curvets with pupae were left in racks inside plastic boxes with water at the bottom to ensure the humidity was high enough that the food disks would not dry out. These boxes were placed in 24° incubators. Vials were checked daily or after weekends for eclosed individuals, these were then sexed through the clear walls of the curvets using a dissecting microscope x10. By scanning the barcode sticker on the lid of the cuvette and a barcode representing their determined sex (e.g. M , F or dead) this information and the date of eclosion could rapidly be entered into the database using a cheap hand held scanner or tablet.


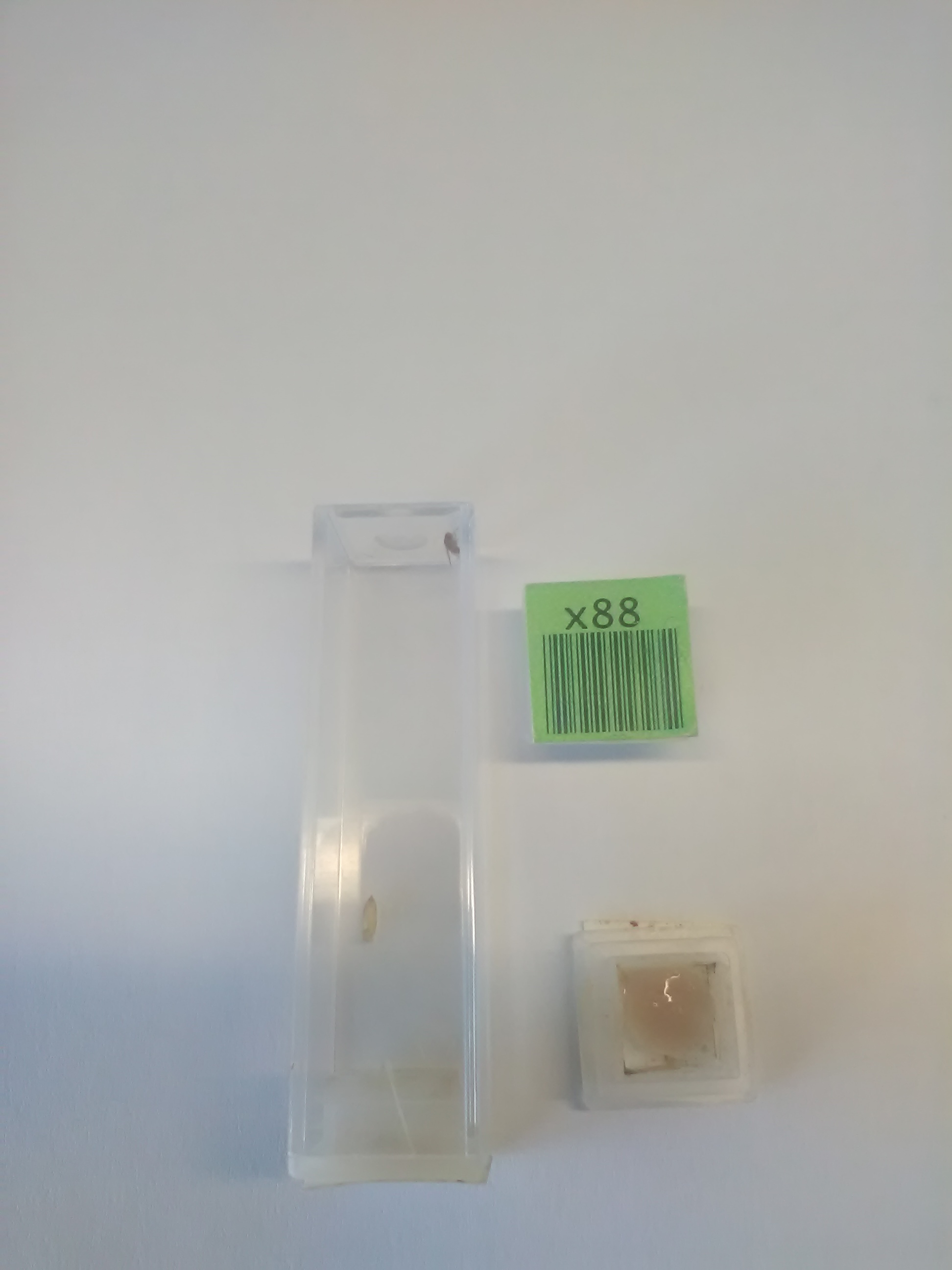


**Left**, cuvette used for eclosion and sexing of individual pupae . Cuvettes are 1x1x4cm and reusable for this purpose (it is better to avoid cuvettes which are opaque on 2 of their vertical surfaces). **Right**, the cuvette lid has a barcode sticker in the outer surface that can be read with a cheap handheld scanner. A disc of filter paper soaked in adult food is placed in the inner part of the lid.

Adults could generally survive 2-3 days in the cuvette. Vials for the next generation were set up using adults after briefly chilling pairs (male and female) of cuvettes on ice and dumping in to a vial with a film. Realistically it is possible for a single user to sex and set up approximately 100 single pair crosses in a day with the help of a database that relates vials to both parents.

By either method crosses can be set up with certain virgins without the need for CO_2_ exposure and mating can commence as soon as possible after the second individual has eclosed. This can save several days over traditional virgin collection techniques.

*Storing phenotyped individuals for future analysis.* The capacity to retrospectively return to any specific phenotyped individual in an experiment is a potentially valuable one. As pupae remain attached to films it is possible to freeze films in individual transparent bags for future analysis. The pupae remain attached even after freezing (-20°) and thawing. The bags do not need to be labeled, as with transparent bags the barcode sticker is clearly visible.


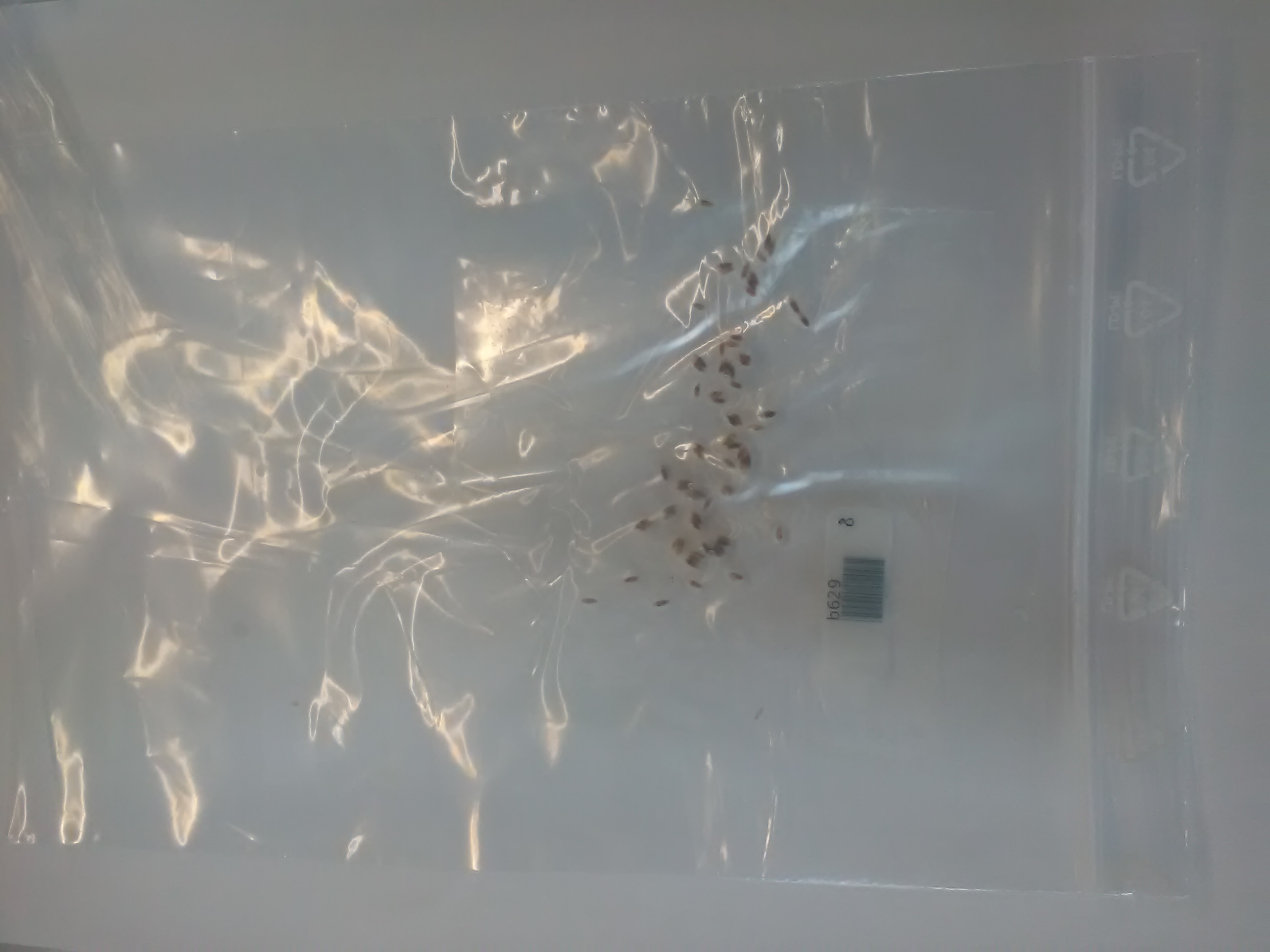


Film in bag used for freezing.

As pairs of adults are generally cleared from vials well before the surface of the food becomes sticky it is easy to recover both parents and freeze them together in labeled tubes.

Both adult and pupae DNA can be reliably extracted from individuals using a variety of standard protocols.

*Costs of establishing an automated phenotyping system.* Assuming that a computer is a available which is suitable to run Cellprofiler (PC, Mac, or Linux) the major cost is equipment to capture images. We used a 350€ digital camera which could be completely controlled from the computer, however a similar system can be established using a tablet should one be available. Illumination was provided by a 30€ A5 electroluminescent light sheet (approximately 30€ posterpoweruk.co.uk or glowhut.com). A light tight box can be custom assembled using a variety of materials (including potentially Lego). Frames to hold films flat can be 3D printed using the files provided (File S2) or custom made. Including the camera our set up cost approximately 500€ (excluding the cost of producing >100 frames). It is also possible that geldoc systems preset in many labs could be also adapted for this purpose.

*Bibliography*

Ashburner M., Golic K., Hawley R. S., 2011 *Drosophila: A Laboratory Handbook*. Cold Spring Harbor Laboratory Press, Cold Spring Harbor, N.Y.

Barron A. B., 2000 Anaesthetising Drosophila for behavioural studies. J. Insect Physiol. **46**: 439–442.
