# Supplementary material for: Automated Phenotyping Indicates Pupal Size in Drosophila Is a Highly Heritable Trait with an Apparent Polygenic Basis
Source: G3 (Bethesda). 2017 Mar 2;7(4):1277–86. doi: 10.1534/g3.117.039883 (PMC5386876; doi:10.1534/g3.117.039883)
Supplement: Supplementary file 8 [file 1277FileS2.zip › File S2/read_me_file_S2.docx]

These files are to print a frame to hold pupae on an overhead film described and File S1.

All files are the same shape just in different formats used for different 3D printing machines.

We have found that black APS plastic works well as printing medium.
