# Supplementary material for: Automated Phenotyping Indicates Pupal Size in Drosophila Is a Highly Heritable Trait with an Apparent Polygenic Basis
Source: G3 (Bethesda). 2017 Mar 2;7(4):1277–86. doi: 10.1534/g3.117.039883 (PMC5386876; doi:10.1534/g3.117.039883)
Supplement: Supplementary file 9 [file 1277FileS3.zip › File S3/readme.pdf]

This document shows you how to set up and use .zipped files to successfully run a test file. It will also show how to try your own .jpg files (though the coin measurement part will probably fail, but it will not throw an error and you should still get meaningful results for everything else)

1) Create a new default output folder and put the Model2016.xml and the coinseed2016.tiff file in it. !Do not specify the same folder the input folder is in or it will overwrite it.
2) Download and install a stable version of Cellprofiler. This pipeline was used on PC V2.1.0 64bit. However I just tried it on the latest version and the pipeline is fine MAC V2.2.0 64bit, have not tried it on beta version V2.3.0.
3) Open the program. File> preferences > set the default output folder to the one which you just made above. All your results will appear here organized in subfolders named based on the input folder name.
4) Drag the file coinseed2016.tiff into the into the window then file>save project. This file will now always appear in the project- it must remain here .
5) Next drag the test .jpg file (P94C.jpg) in the folder 2017_01_01. This is how you will generally add files to be analyzed. Press the Analyze Images button on the lower left of the window.

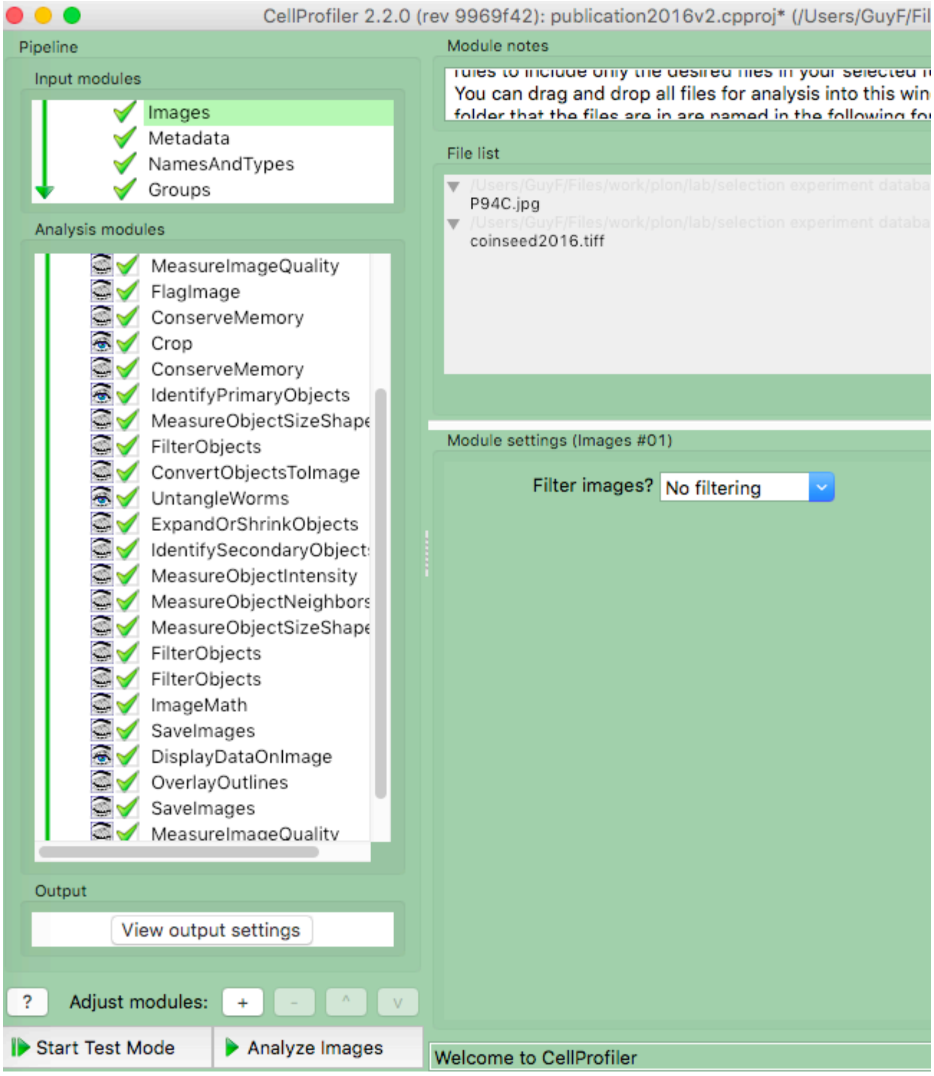

Note that in this pipeline input files must have .jpg (lowercase) to be analyzed. If you files does not have this change the options in the 'images' metadata and NamesAndTypes windows.

To remove analyzed files from the window right click or control
There will be a new set of output files created for each folder i.e. if you have 200 .jpg files in a folder you will get 1 set of .xls files with data for all 200. You will also get a cropped image and an overlaid image for each of the input files. All files will be named and organized in a logical manner.
The pipeline used is identical for all analysis parameters to that used in the publication, but the version here has simpler file naming requirements and the locations of the files Model2016.xml and the coinseed2016.tiff are different.

I have added a few notes on every module and highlighted key parameters. The help within Cellprofiler is very good and contextual and available by pressing the question mark button in the lower left part of the window or next to the parameter in question.

The test mode is a very useful function if you need to change parameters.

http://d1zymp9ayga15t.cloudfront.net/CPmanual/Help_Testing_Your_Pipeline.html

If you click on to open the eyes on more modules you can work out where you are having problems and use the test mode and help to fix them.

Obviously analysis is easier if you have a high contrast between background and pupae to start with so try to set your photographs up to do this.

The pipeline is set up so that a 1cent Ç coin which is 16.25mm in diameter is approximately 434 pixels in diameter. If your photos are within 7% of this scale the pipe line should work without modifications. Feel free to contact me if you are having problems.
