# Supplementary figures and images for: Automated Phenotyping Indicates Pupal Size in Drosophila Is a Highly Heritable Trait with an Apparent Polygenic Basis
Source: G3 (Bethesda). 2017 Mar 2;7(4):1277–86. doi: 10.1534/g3.117.039883 (PMC5386876; doi:10.1534/g3.117.039883)

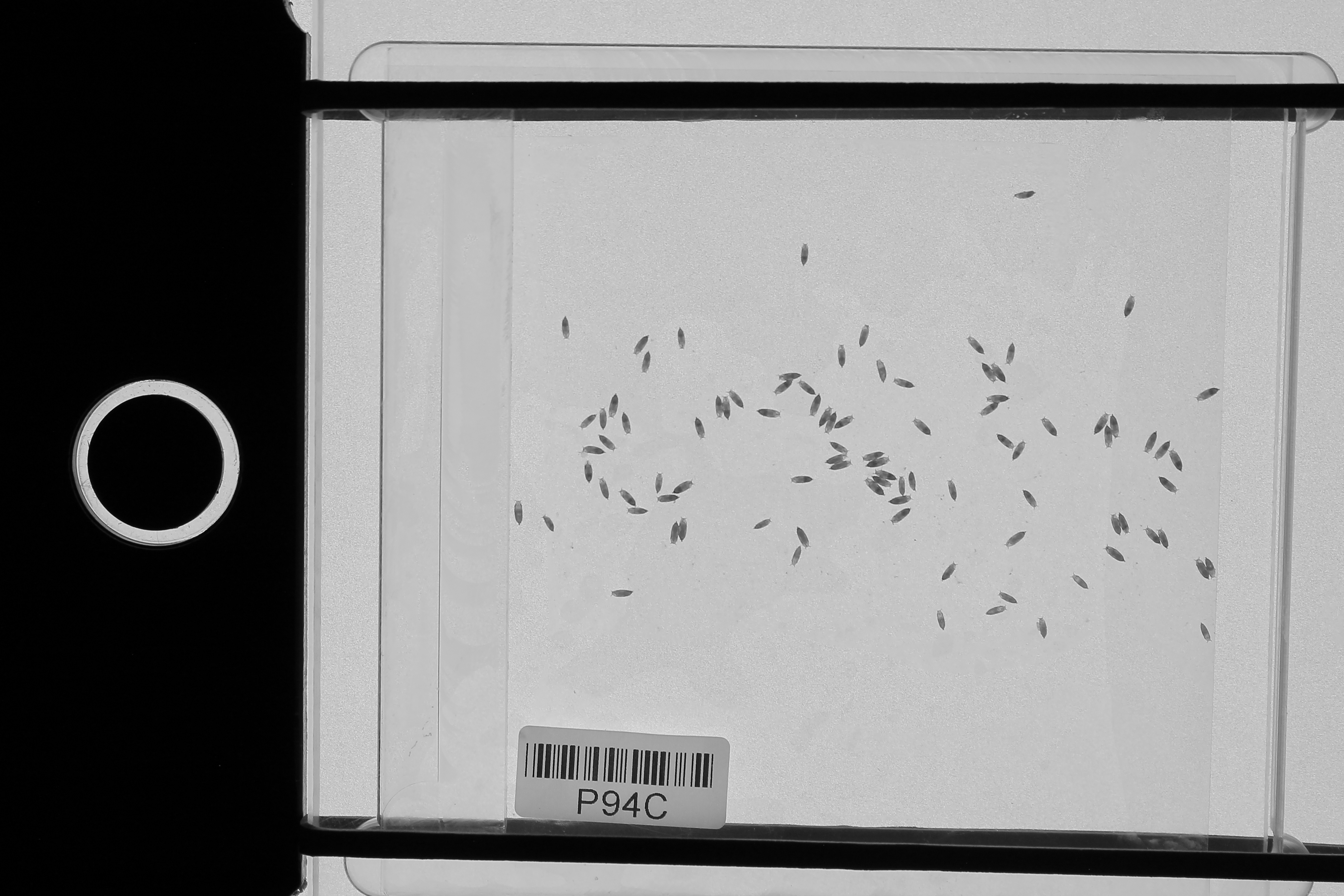

Supplement: Supplementary file 9 [file 1277FileS3.zip › File S3/2017_01_01/P94C.jpg]

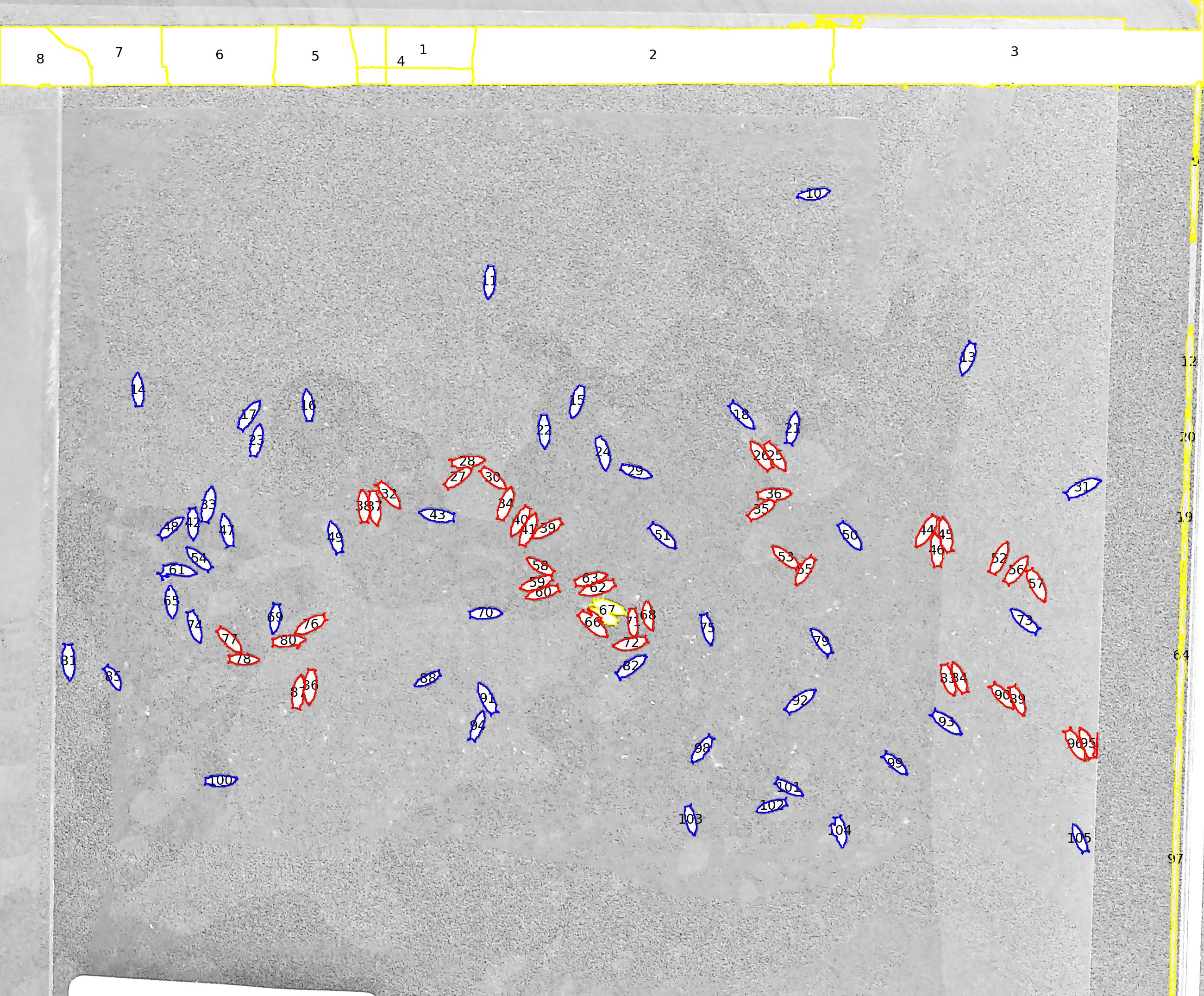

Supplement: Supplementary file 9 [file 1277FileS3.zip › File S3/output/2017_01_01 expected results/all_outlines/P94C_outlines.jpeg]

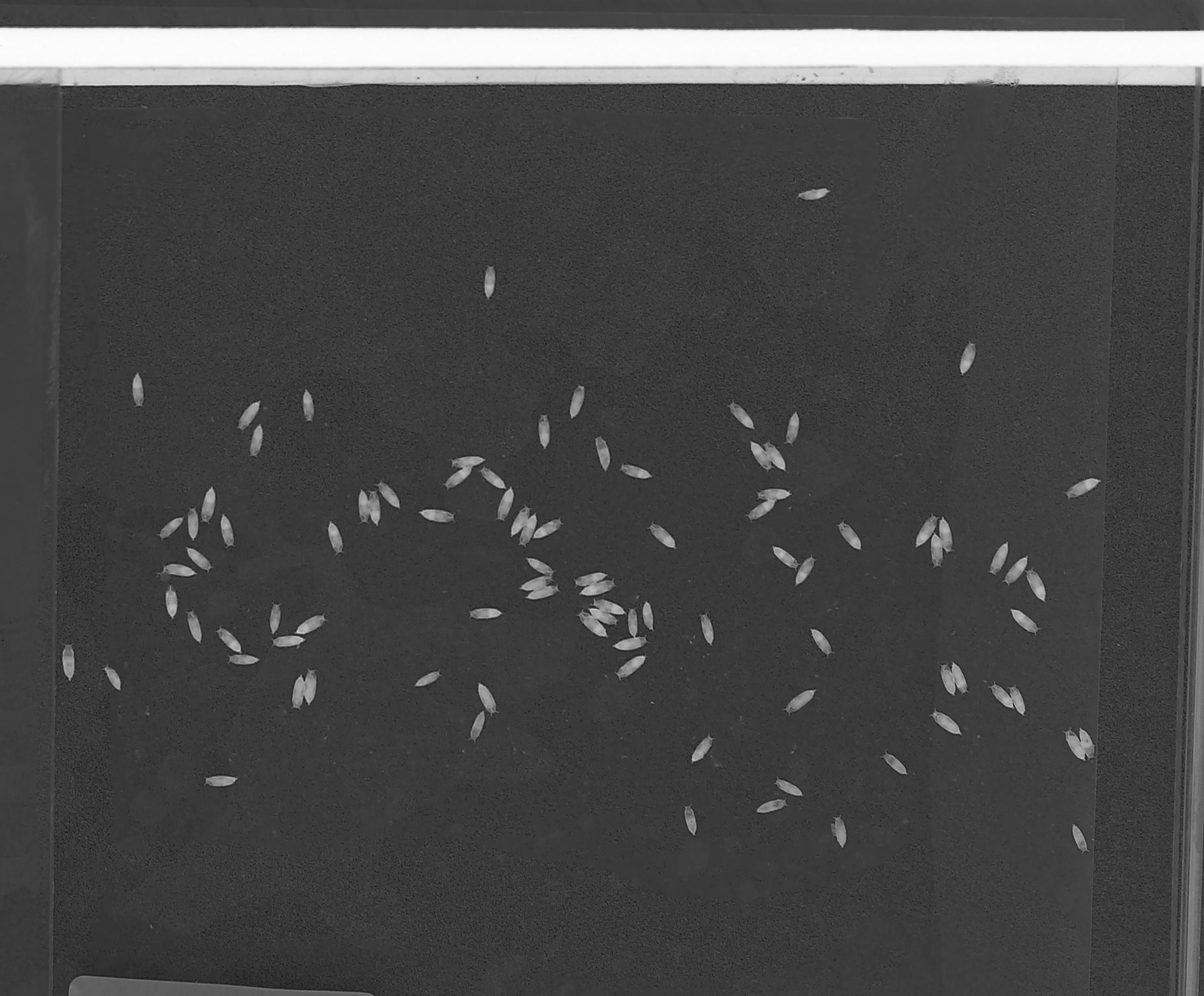

Supplement: Supplementary file 9 [file 1277FileS3.zip › File S3/output/2017_01_01 expected results/crop/P94C_croped.jpeg]

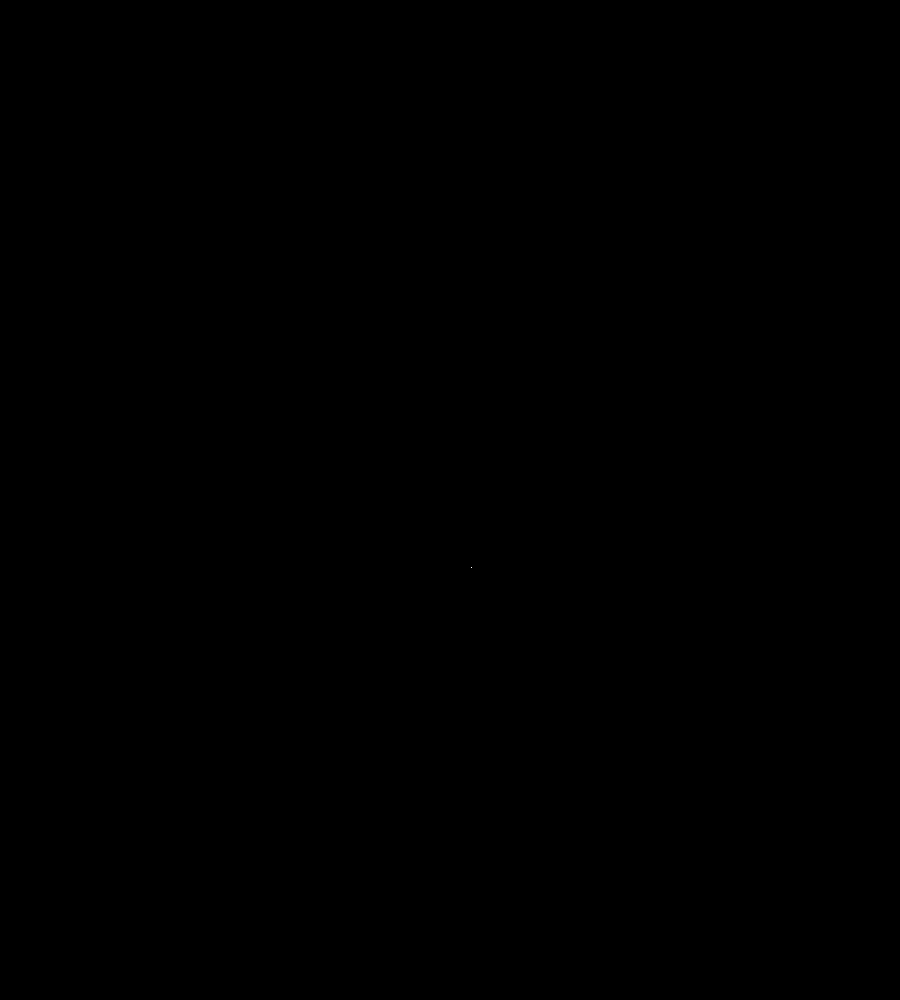

Supplement: Supplementary file 9 [file 1277FileS3.zip › File S3/output/coinseed2016.tiff]
