# Supplementary material for: Automated Phenotyping Indicates Pupal Size in Drosophila Is a Highly Heritable Trait with an Apparent Polygenic Basis
Source: G3 (Bethesda). 2017 Mar 2;7(4):1277–86. doi: 10.1534/g3.117.039883 (PMC5386876; doi:10.1534/g3.117.039883)
Supplement: Supplementary file 10 [file 1277FileS4.zip › File S4/Read_me_S4.docx]

Figure 1A

File S4

((NOT vialPUBLICATION_control ="329") AND (NOT vialPUBLICATION_control ="335" ) AND (vialvial_included_count >= 15) AND (vialPUBLICATION_purpose ="broad") AND (RILcount_greater_15>=3))

OR

((NOT vialPUBLICATION_control ="329") AND (NOT vialPUBLICATION_control ="335" ) AND (vialvial_included_count>= 15) AND (vialPUBLICATION_purpose ="narrow" ))

Y axis = frequency

X axis = mm_included_MajorAxisLength

Figure3

File S4

(reverse::mm_rev_len>0 )

Y axis = reverse::mm_rev_len

X axis = mm_included_MajorAxisLength

GET slope and R2

GUIDE: axis(dim(1), label("mm_included_MajorAxisLength"))

GUIDE: axis(dim(2), label("reverse::mm_rev_len"))

Figure 6A

File S4

((NOT PUBLICATION_control ="329") AND (NOT PUBLICATION_control ="335" )AND (vial_included_count >= 15) AND (PUBLICATION_purpose ="narrow" ))

Y axis = cumulative percentage

X axis = ABS(Vial_included_count-65))*0.02 is absolute difference

Figure 6B

File S4

Data:((NOT PUBLICATION_control ="329") AND (NOT PUBLICATION_control ="335" )AND (vial_included_count >= 15) AND (PUBLICATION_purpose ="broad" AND RILcount_greater_15>=3))

Y axis = cumulative percentage

X axis = corrected_length_abs_diff =ABS(Vial_included_count-65)*0.02)

Fig 7

File S4

select ( vial_included_count= 51..60)

Ran with pupae re-sampling script

| **Field name** | **Explanation** |
| --- | --- |
| BARCODE_vial | Unique ID for vial  (database primary key) |
| manual_count_number | Count of true number of pupae photographed based on manual count. Minority of vials have values. |
| mm_parent_father | Length in mm of vial father. Single pair crosses only |
| mm_parent_midpoint | (mm_parent_father + mm_parent_mother)/2 |
| mm_parent_mother | Length in mm of vial mothe. Single pair crosses only |
| mm_vial_ave | Mean pupal length of all measured puape mm |
| mm_vial_ave_greater15 | Mean pupal length of all measured puape mm- only calculated if ‘vial_included_count ≥15’ |
| PUBLICATION_control | Value present if vial was measurement control. Two stocks were consistently used throughout experiment 329 and 335 See Table S1.  Generally excluded from all analysis. |
| PUBLICATION_dataset | Either 8_way or 4_way |
| PUBLICATION_purpose | Dataset ‘narrow’ or ‘broad’ used for calculation of respective heritability’s |
| vial_included_count | Number of pupae measured by automated system (does not include pupae manually excluded) |
| files::photo_date_week | Week on which vial was photographed adjusted to run between years. |
| parents_8_way_narrow_cross::cross_parent_father_ID | Unique ID for father  ‘BARCODE_vial & pupae object number’ |
| parents_8_way_narrow_cross::cross_parent_father_ID_dspr | DSPR ID of father single pair 8_way crosses only  http://wfitch.bio.uci.edu/~dspr/riltable/index.html |
| parents_8_way_narrow_cross::cross_parent_mother_ID | Unique ID for mother  ‘BARCODE_vial & pupae object number’ |
| parents_8_way_narrow_cross::cross_parent_mother_ID_dspr | DSPR ID of mother single pair 8_way crosses only  http://wfitch.bio.uci.edu/~dspr/riltable/index.html |
| parents_8_way_narrow_cross::lookup_father | Vial average for vial father came from  single pair 8_way crosses only |
| parents_8_way_narrow_cross::lookup_mother | Vial average for vial father came from  single pair 8_way crosses only |
| parents_8_way_narrow_cross::narrow_8_way_duplicate_set | 6 of the 67 ‘8_way narrow crosses’ are duplicate. Duplicate pairs are identified in this field |
| parents_8_way_narrow_cross::RIL_midpoint_mm | (parents_8_way_narrow_cross::lookup_father + parents_8_way_narrow_cross::RIL_midpoint_mm)/2 |
| RIL::count_greater_15 | Count of RIL repeat measurements where ‘vial_included_count ≥15’ |
| RIL::DSPR_stock | ID of RIL stocks  Stocks 0-100 MPI Plön stocks  >100 DSPR stocks http://wfitch.bio.uci.edu/~dspr/riltable/index.html |
| RIL::mm_RIL_average_using_vialsGreater15 | RIL pupal average length  Using only vials ‘vial_included_count ≥15’ |
| RIL::RIL_average | RIL pupal average length |
| **AreaShape_MajorAxisLength** | Pupal length in pixels (uncorrected by coin) |
| Children_pupaeSECONDARYobjectsFILTEREDhigh_Count | If =1 pupa is considered to be high confidence (blue outline) |
| **Children_pupaeSECONDARYobjectsFILTEREDlower_Count** | If =1  and Children_pupaeSECONDARYobjectsFILTEREDhigh_Count =0 pupa is considered to be medium confidence (red outline) See file S1 |
| **corrected_length_abs_diff** | Absolute difference between actual pupal measurement and that corrected for vial density. See equation in text.  Abs((‘vial::vial_included_count_greater15’-65)*-.002) |
| **mm_included_MajorAxisLength** | Puapl length in mm corrected by coin measurement |
