# Supplementary material for: Automated Phenotyping Indicates Pupal Size in Drosophila Is a Highly Heritable Trait with an Apparent Polygenic Basis
Source: G3 (Bethesda). 2017 Mar 2;7(4):1277–86. doi: 10.1534/g3.117.039883 (PMC5386876; doi:10.1534/g3.117.039883)
Supplement: Supplementary file 11 [file 1277FileS5.zip › File S5/Read_Me_S5.docx]

Figure 1B

File S5

Data:((NOT PUBLICATION_control ="329") AND (NOT PUBLICATION_control ="335" )AND (vial_included_count >= 15) AND (PUBLICATION_purpose ="broad" AND RILcount_greater_15>=3))

OR

((NOT PUBLICATION_control ="329") AND (NOT PUBLICATION_control ="335" )AND (vial_included_count >= 15) AND (PUBLICATION_purpose ="narrow" ))

Y axis = frequency

X axis = mm_vial_ave_greater15

Figure 4

File S5

Data: (manual_count_number >0)

Y axis = vial_included_count

X axis = manual_count_number

estimated vial density (automated count photographed puape)

Figure 5

File S5

Data: (PUBLICATION_control ="329") OR (PUBLICATION_control ="335" )AND (vial_included_count >= 15)

Y axis = mm_vial_ave_greater15

X axis = vial_included_count

Fig 8

File S5

select ((DSPRcount_greater_15>=12= )AND (vial_included_count >= 15)) OR ((PUBLICATION_control ="329") OR (PUBLICATION_control ="335" )AND (vial_included_count >= 15) AND (RILcount_greater_15>=12))

Ran with vial re-sampling script

Figure 9

File S5

8_way line green closed circles

((NOT PUBLICATION_control ="329") AND (NOT PUBLICATION_control ="335" )AND (vial_included_count >= 15) AND (PUBLICATION_purpose ="narrow" ) AND (PUBLICATION_dataset ="8-way"))

Y axis = mm_vial_ave_greater15

X axis = parents_8_way_narrow_crossRIL_midpoint_mm

4_way line blue open circles

((NOT PUBLICATION_control ="329") AND (NOT PUBLICATION_control ="335" )AND (vial_included_count >= 15) AND (PUBLICATION_purpose ="narrow" ) AND (PUBLICATION_dataset="4_way"))

Y axis = mm_vial_ave_greater15

X axis = mm_parent_mid

4-way narrow due to recombination all siblings on a vial are not genetically identical, consequently in calculating the mid parent value it is most appropriate to use the (actual length of the father + actual length of the mother)/2= **mm_parent_midpoint**

However in the 8-way narrow estimate which are crosses between RILs it can reasonably be assumed that all the siblings in a vial are genetically identical, consequently it is reasonable to use the vial average from which the parents are taken to calculate the mid parent the )(mean length vial from which the father was taken + mean length vial from which the mother was taken)/2=parents_8_way_narrow_crossRIL_midpoint_mm

). The values given in table 2 are calculated in this manner.

If the values in table 2 are calculated using the actual lengths of mother and father ( as for the 4-way narrow) the values are h^2^=0.41 ± 0.08SE and R^2^= 0.29 (which are slightly lower than those presented in Table 2 of h^2^=0.50 ± 0.09SE and R^2^= 0.33)

| **Field name** | **Explanation** |
| --- | --- |
| BARCODE_vial | Unique ID for vial  (database primary key) |
| manual_count_number | Count of true number of pupae photographed based on manual count. Minority of vials have values. |
| mm_parent_father | Length in mm of vial father. Single pair crosses only |
| mm_parent_midpoint | (mm_parent_father + mm_parent_mother)/2 |
| mm_parent_mother | Length in mm of vial mothe. Single pair crosses only |
| mm_vial_ave | Mean pupal length of all measured puape mm |
| mm_vial_ave_greater15 | Mean pupal length of all measured puape mm- only calculated if ‘vial_included_count ≥15’ |
| PUBLICATION_control | Value present if vial was measurement control. Two stocks were consistently used throughout experiment 329 and 335 See Table S1.  Generally excluded from all analysis. |
| PUBLICATION_dataset | Either 8_way or 4_way |
| PUBLICATION_purpose | Dataset ‘narrow’ or ‘broad’ used for calculation of respective heritability’s |
| vial_included_count | Number of pupae measured by automated system (does not include pupae manually excluded) |
| files::photo_date_week | Week on which vial was photographed adjusted to run between years. |
| parents_8_way_narrow_cross::cross_parent_father_ID | Unique ID for father  ‘BARCODE_vial & pupae object number’ |
| parents_8_way_narrow_cross::cross_parent_father_ID_dspr | DSPR ID of father single pair 8_way crosses only  http://wfitch.bio.uci.edu/~dspr/riltable/index.html |
| parents_8_way_narrow_cross::cross_parent_mother_ID | Unique ID for mother  ‘BARCODE_vial & pupae object number’ |
| parents_8_way_narrow_cross::cross_parent_mother_ID_dspr | DSPR ID of mother single pair 8_way crosses only  http://wfitch.bio.uci.edu/~dspr/riltable/index.html |
| parents_8_way_narrow_cross::lookup_father | Vial average for vial father came from  single pair 8_way crosses only |
| parents_8_way_narrow_cross::lookup_mother | Vial average for vial father came from  single pair 8_way crosses only |
| parents_8_way_narrow_cross::narrow_8_way_duplicate_set | 6 of the 67 ‘8_way narrow crosses’ are duplicate. Duplicate pairs are identified in this field |
| parents_8_way_narrow_cross::RIL_midpoint_mm | (parents_8_way_narrow_cross::lookup_father + parents_8_way_narrow_cross::RIL_midpoint_mm)/2 |
| RIL::count_greater_15 | Count of RIL repeat measurements where ‘vial_included_count ≥15’ |
| RIL::DSPR_stock | ID of RIL stocks  Stocks 0-100 MPI Plön stocks  >100 DSPR stocks http://wfitch.bio.uci.edu/~dspr/riltable/index.html |
| RIL::mm_RIL_average_using_vialsGreater15 | RIL pupal average length  Using only vials ‘vial_included_count ≥15’ |
| RIL::RIL_average | RIL pupal average length |
